# Integrated Microfluidic Chip for Neutrophil Extracellular Vesicle Analysis and Gastric Cancer Diagnosis

**DOI:** 10.1021/acsnano.4c16894

**Published:** 2025-03-10

**Authors:** Dan Yu, Jianmei Gu, Jiahui Zhang, Maoye Wang, Runbi Ji, Chunlai Feng, Hélder A. Santos, Hongbo Zhang, Xu Zhang

**Affiliations:** † Jiangsu Key Laboratory of Medical Science and Laboratory Medicine, School of Medicine, Jiangsu University, Zhenjiang, Jiangsu 212013, China; ‡ Department of Clinical Laboratory Medicine, Affiliated Tumor Hospital of Nantong University, Nantong, Jiangsu 226361, China; § School of Pharmacy, Jiangsu University, Zhenjiang, Jiangsu 212013, China; ∥ Department of Biomaterials and Biomedical Technology, The Personalized Medicine Research Institute (PRECISION), University Medical Center Groningen, Groningen 9713 AV, Netherlands; ⊥ Pharmaceutical Sciences Laboratory, Åbo Akademi University, Turku 20520, Finland; # Turku Biosciences Center, University of Turku and Åbo Akademi University, Turku 20520, Finland

**Keywords:** extracellular vesicles, neutrophils, microfluidic chip, biomarker, gastric cancer

## Abstract

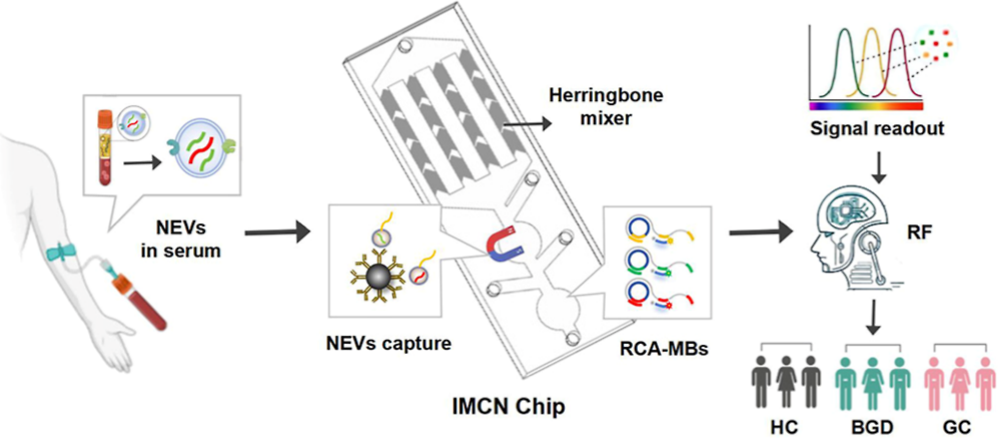

Neutrophil-derived extracellular vesicles (NEVs) are critically involved in disease progression and are considered potential biomarkers. However, the tedious processes of NEV separation and detection restrain their use. Herein, we presented an integrated microfluidic chip for NEV (IMCN) analysis, which achieved immune-separation of CD66b^+^ NEVs and multiplexed detection of their contained miRNAs (termed NEV signatures) by using 10 μL serum samples. The optimized microchannel and flow rate of the IMCN chip enabled efficient capture of NEVs (>90%). After recognition of the captured NEVs by a specific CD63 aptamer, on-chip rolling circle amplification (RCA) reaction was triggered by the released aptamers and miRNAs from heat-lysed NEVs. Then, the RCA products bound to molecular beacons (MBs), initiating allosteric hairpin structures and amplified “turn on” fluorescence signals (RCA-MB assay). Clinical sample analysis showed that NEV signatures had a high area under curve (AUC) in distinguishing between healthy control (HC) and gastric cancer (GC) (0.891), benign gastric diseases (BGD) and GC (0.857). Notably, the AUC reached 0.912 with a combination of five biomarkers (NEV signatures, CEA, and CA199) to differentiate GC from HC, and the diagnostic accuracy was further increased by using a machine learning (ML)-based ensemble classification system. Therefore, the developed IMCN chip is a valuable platform for NEV analysis and may have potential use in GC diagnosis.

## Introduction

Gastric cancer (GC) is the third leading cause of cancer-related death worldwide.^1^ As a result of atypical symptoms and rapid development at the onset, GC patients are often diagnosed at a late stage, leading to a low five-year survival rate and poor prognosis. Carcinoembryonic antigen (CEA) and carbohydrate antigen 199 (CA199) are two commonly used serum tumor biomarkers for GC diagnosis but are challenged by low sensitivity and specificity. Extracellular vesicles (EVs) are membrane nanovesicles secreted by almost all types of cells.^2^ The bioactive molecules selectively packaged into EVs are considered promising biomarkers for cancer liquid biopsy.^3−5^ However, the diagnostic accuracy of EVs is limited by their heterogeneity. Increasing studies suggest that profiling EVs of a specific origin is able to reduce the heterogeneity and further increase their diagnostic accuracy.^6−8^

Neutrophils are a critical component of the tumor microenvironment (TME) and play important roles in tumor progression by direct release of bioactive molecules or EV-mediated cellular communications.^9−12^ Neutrophils are reported to be the main sources of elevated cell free DNA (cfDNA) in the plasma of cancer patients.^13^ Previous studies suggested that a high density of CD66b^+^ neutrophils and neutrophil/lymphocyte ratio (NLR) is closely associated with tumor progression and patient survival.^14^ CD66b was also previously identified as one of the most sensitive and specific markers to detect neutrophil-derived EVs (NEVs).^15^ Therefore, CD66b^+^ NEVs may offer new information for cancer liquid biopsy. We previously reported that the abundance of certain miRNAs (miR-223-3p and miR-425-5p) is increased in the serum CD66b^+^ NEVs of GC patients and offer potential biomarkers for GC diagnosis.^16^ However, it remains technically challenging to selectively separate NEVs and accurately analyze their cargos. Conventional EV isolation approaches, such as ultracentrifugation (UC), ultrafiltration, size-exclusion chromatography (SEC), and precipitation techniques, need time-consuming processes and obtain EVs with relatively low purity.^17^ In addition, the existing technologies for EV cargo detection, such as qRT-PCR and ELISA, involve multiple manual steps and take a long assay time.^18,19^ Moreover, these analyses are prone to be interfered with free nucleic acids and proteins in the biofluids, whose abundance is several orders higher than those in EVs. Therefore, developing a rapid, simplified, and robust platform to separate, quantify, and profile EVs in the biofluids is essential to promote their clinical use.

Microfluidic chips have been used in EV separation and detection.^20,21^ Compared with conventional EV isolation approaches, microfluidic chips exhibit the superiority of easy-to-operate, small sample volume demand, and high yield.^22^ The specific surface proteins are generally used to identify unique EV subsets.^23^ By manipulating the affinity particles/magnetic beads or modifying the microchannel surface with antibodies or aptamers,^24−26^ microfluidic chips can efficiently and specifically separate EV subsets. Moreover, microfluidic chips enable in situ detection of EV cargos by integrating colorimetric,^27^ fluorescent, or electrochemical detection technologies,^28,29^ skipping the laborious and time-consuming procedures of traditional techniques. For example, Li et al. have developed an anti-CD9 antibody-coated 3D porous sponge microfluidic chip integrating quantum dot (QD)-labeled SORL1 (sorting protein-related receptor) antibody, which enables a high capture of serum EVs (90%) and rapid quantification of SORL1 on them.^30^ Lu et al. have presented an integrated microfluidic EV isolation and detection system (EXID system). By incorporating CD9 antibody-modified magnetic beads and PD-L1 protein recognition probes, the EXID system achieves the rapid separation and quantification of PD-L1^+^ EVs within 2 h.^31^ The simultaneous detection of multiple EV cargoes, such as proteins and microRNAs (miRNAs), may improve the accuracy of diagnosis. For example, Zhou et al. have designed a 3D microfluidic chip that incorporates immune capture and QDs labeling with molecular beacon (MB) detection to achieve a comprehensive profiling of EV proteins and miRNAs, which shows an overall accuracy of around 100% for cancer diagnosis and staging.^28^ Therefore, the microfluidic platform that integrates rapid separation and sensitive and multiplexed detection of EVs will offer a promising tool for cancer liquid biopsy.

Here, we reported an IMCN analysis, which favored rapid separation of NEVs and triplex detection of CD66b^+^ NEV abundance and their contained miRNAs (miR-223-3p and miR-425-5p), which were termed NEV signatures. CD66b antibody-coupled Dynabeads were prepared to specifically recognize and sort NEVs, and the CD63 aptamers were simultaneously introduced for the recognition of NEVs, thereby enabling the separation and quantification of NEVs. The miRNAs in NEVs were obtained by thermal lysis on chip, which is simple and rapid for subsequent miRNA detection and is free from the lengthy processes of RNA extraction and reverse transcription. RCA-MB assay was established and optimized for the amplification and detection of NEV signatures, which was carried out under isothermal conditions, avoiding the expensive reagents, special equipment, and strict temperature control procedures of qPCR. We tested the levels of NEV signatures in serum samples from healthy controls (HCs), benign gastric diseases (BGD), and GC patients and analyzed the performance of NEV signatures alone or combined with conventional serum biomarkers. Moreover, a random forest (RF)-assisted classification system was established to improve the diagnostic accuracy and promote their clinical use. The IMCN chip enabled sensitive quantification of NEVs and their contained miRNAs (NEV signatures) in less than 4 h by using only 10 μL of serum samples. We expected that the developed IMCN chip would provide a novel tool for NEV analysis and GC diagnosis.

## Results and Discussion

### Design Principle of IMCN Chip

We established the IMCN chip model using SolidWorks software. The microfluidic device was a polydimethylsiloxane (PDMS) chip, which is 85 mm × 30 mm in size and composed of five holes (3 mm in diameter), four sets of herringbone mixer channels, a magnetic capture chamber (chamber 1, 10 mm in diameter), and a reaction chamber (chamber 2, 10 mm in diameter) (Figure S1A). Each mixer channel had four groups of asymmetrical herringbone grooves, and each group contained 20 grooves. The angle between the herringbones and channel axis was set at (θ) 45° (Figure S1B,C). The channel aspect ratio is one of the key factors that affects fluid mixing. It has been reported that channels with deeper grooves always obtain higher fluid mixing efficiency. In addition, the wider flow channel helps generate more vortex and turbulent structures and enhance the transfer of mass and momentum between fluids.^32^ Therefore, the appropriate aspect ratio is conducive to the formation of three-dimensional flow characteristics and promotes the fluid to get fully mixed in all directions. We performed computational fluid dynamics (CFD) simulation to analyze the fluid dynamics of the chip with different aspect ratios (80:40, 80:50, 100:40, and 100:50 μm). As a result, the fluid flow formed more complex vortex structures at a 100:50 μm channel aspect ratio. Streamline charts and pressure profiles were applied to evaluate the fluid characteristics in channels with different parameters. As shown in Figure S1D, streamline charts indicated that the flow velocity on the sides of the fluid was much higher than that in the middle when the channel was set at a 100:50 μm width-to-depth ratio, and the pressure profile validated that the highest-pressure drop was obtained at this ratio (Figure S1E). Consequently, the particles in the sample tended to move into the region of high velocity, which could increase the collision frequency between NEVs and Dynabeads and effectively improve the NEVs capture efficiency.

## Working Principle of IMCN Chip

**Figure 1:**
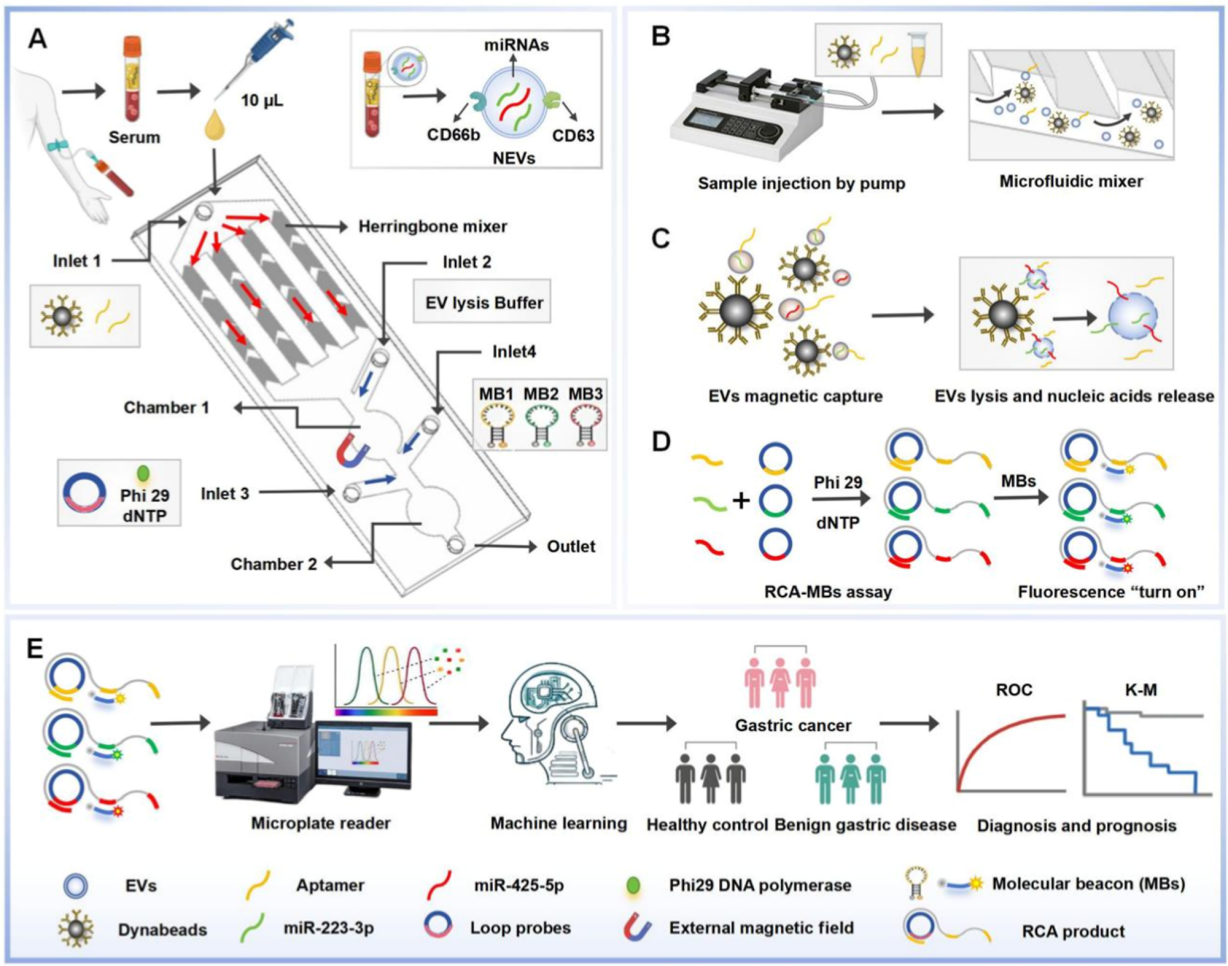
Schematic illustration of IMCN chip for NEV separation and detection. (A) The IMCN chip integrates the NEV separation, enrichment, lysis, and signal amplification and detection. Serum samples were injected from “inlet 1”, and the NEVs were enriched and lysed in “chamber 1”. After introducing LP, Phi29 DNA polymerase, dNTP mix, and MBs, the RCA-MB assay was reacted in “chamber 2”, and the reaction solution was collected at “outlet” for signal reading. (B) NEVs capturing by CD66b antibody-coupled Dynabeads and aptamer recognition in herringbone micromixer. (C) NEV lysis and release of aptamers and contained miRNAs. (D) Working principle of RCA-MB assay. (E) Fluorescence signal detection by microplate read system, and the data analysis via machine learning for GC diagnosis and prognosis. Some elements of the figure were provided by BioRender.

The workflow of the IMCN chip had five steps (Figure 1A). First, 10 μL of serum samples, together with CD66b antibody-coupled Dynabeads and CD63 aptamers, was driven through “inlet 1”. With the constant thrust of the injection pump (Figure S2A), NEVs were labeled with CD63 aptamers and captured by CD66b antibody-coupled Dynabeads in herringbone-structured microchannels (Figure 1B). After NEVs enrichment in “chamber 1”, the chip channels were sequentially washed with PBS and air at a flow rate of 10 μL/min for 10 min to remove wastes and impurities and ensure no remaining Dynabeads and complete NEV collection (Figure S2B–D). Second, the NEVs enriched by an external magnetic field were then lysed with heated DEPC water (95 °C) to release CD63 aptamers and miRNAs in NEVs (Figure 1C). Third, the released CD63 aptamers and miRNAs in NEVs were used as primers to hybridize with three prepared loop probes (LP) and trigger triplex RCA reactions with the assistance of Phi29 DNA polymerase and dNTPs. Fourth, three MBs labeled with Cy3, FAM, and Cy5 were introduced and reacted with the RCA products, breaking the “turned off” state of MBs that was induced by the proximity of fluorophores and the quenching groups and initiating allosteric hairpin structures and amplified “turn on” fluorescence signals (RCA-MB assay; Figure 1D). Finally, the reaction solutions were collected and measured by the cell imaging microplate detection system, and the readouts were further analyzed by a machine learning algorithm for diagnostic performance evaluation (Figure 1E).

### Characterization of NEVs Isolated by Ultracentrifugation

NEVs isolated by UC from a neutrophil cell culture medium (NCM) were characterized and used for chip performance evaluation. We first characterized the properties of NEVs by transmission electron microscopy (TEM), nanoparticle tracking analysis (NTA), and Western blot. The results showed that UC-isolated NEVs had typical cup-shaped morphology, uniform particle size (∼140 nm), and high expression of EV markers (Figure S3A–C). In addition, the expression and location of CD66 on NEVs were also characterized. As shown in Figure S3D, CD66b was detected on the membrane of NEVs by gold-labeled immune-TEM, and the colocalization of CD66b and CD63 on NEVs was further verified by immunofluorescence microscope (Figure S3E). These results confirmed the high purity of UC-isolated NEVs and their utility for the downstream chip performance analysis.

### Characterization of Aptamer Binding Activity

Before the on-chip assay, we validated the binding activity of aptamers to NEVs. FAM-labeled CD63 aptamers (green) and Dil-stained NEVs (red) were coincubated on the slides, and the binding was observed under a laser confocal fluorescence microscope. As shown in Figure S4A, the green and red fluorescence were found highly colocalized, suggesting that the aptamers efficiently bound to CD63 on NEVs.

For further optimization, different concentrations of FAM-labeled CD63 aptamers were incubated with NEVs that were covalently coupled to aldehyde beads, and the fluorescence intensity was then detected by flow cytometry. As shown in Figure S4B,C, the fluorescence intensity gradually increased with the increase of CD63 aptamer concentration and reached the highest level when 1 μM aptamer was used. The fluorescence intensity did not continue to increase when more aptamers were added, and around 70% of NEVs were labeled when 1 μM CD63 aptamer was used (Figure S4D). Therefore, 1 μM was chosen as the optimal aptamer concentration.

In addition, to verify the stability and applicability of aptamers to label NEVs from serum samples, the aptamers were incubated with 10% PBS diluted serum samples (10 μL of serum was diluted ten times by 90 μL of PBS) at 37 °C for 3 h with an interval of 0.5 h or heated at 95 °C for 10 min. After that, the aptamers were applied for agarose gel electrophoresis, and the results showed that they were stable with the extension of time, suggesting good stability and resistance to the degradation by nuclease in serum samples (Figure S5A) and the high-temperature environment (Figure S5B).

### Evaluation of the Capturing Performance of IMCN Chip by Using UC-Isolated NEVs

**Figure 2:**
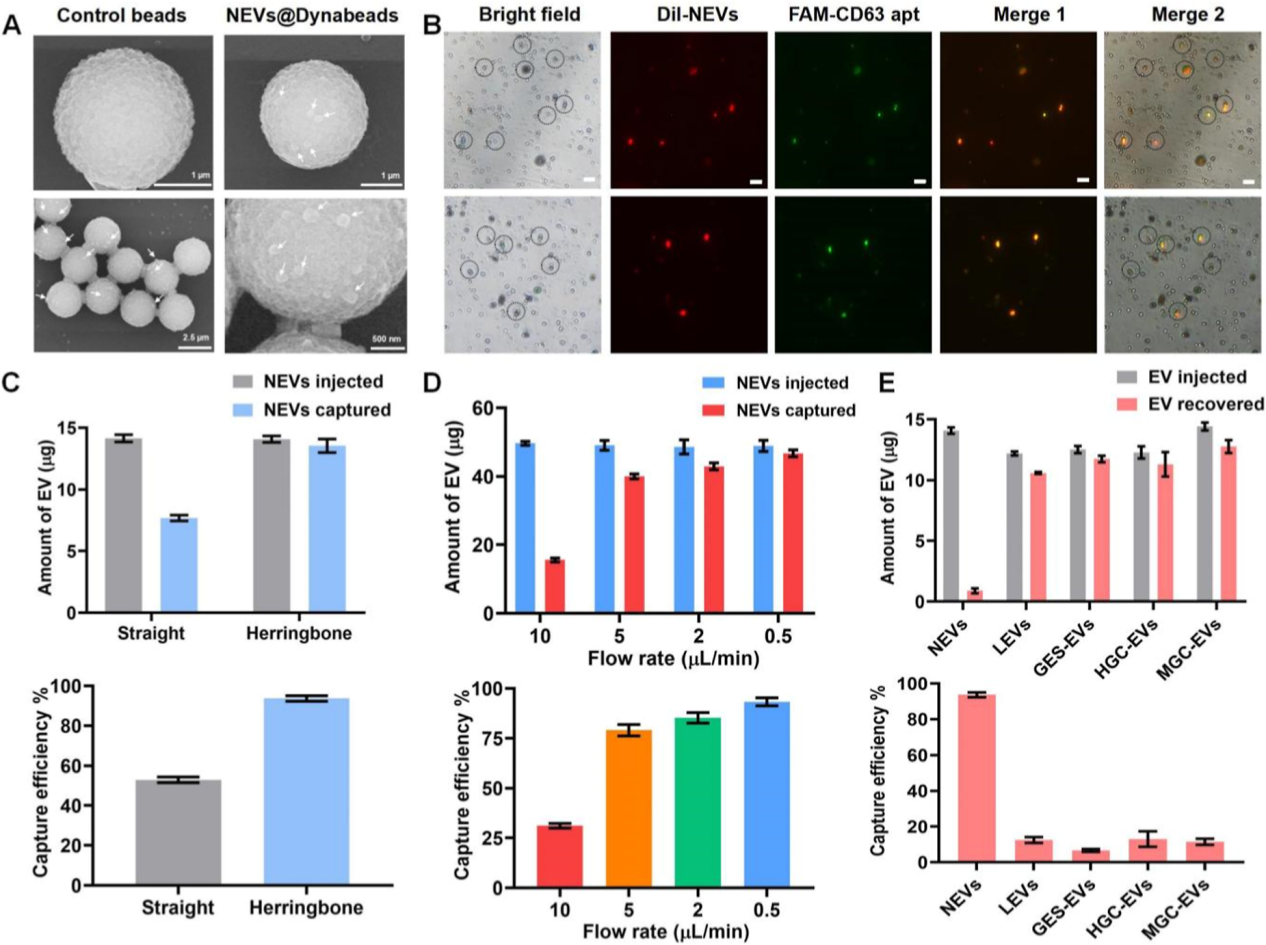
Evaluation of the capturing performance of IMCN chip by using UC-isolated NEVs. (A) SEM images of the control beads (top left) and NEVs captured beads (NEVs@Dynabeads). The captured NEVs were pointed with white arrow. (B) Characterization of the aptamer labeling (green) to the captured Dil-stained NEVs (red) under fluorescence microscope (scale bar, 5 μm). The Dynabeads with captured NEVs and aptamer labeling were circled with black dotted line. (C) The capturing performance of chips with straight channels and herringbone channels on NEVs. (D) Optimization of the chip flow rates (from 10 to 0.5 μL/min) for NEVs capturing. (E) The specificity of the chip for NEVs capturing (NEVs, neutrophil-derived EVs; LEVs, lymphocyte-derived EVs; GES-EVs, human gastric epithelial cell-derived EVs; HGC-EVs and MGC-EVs, human GC cell-derived EVs).

NEVs isolated by UC were used for chip performance evaluation and optimization. First, NEVs and CD66b antibody-coupled Dynabeads were pumped into the IMCN chip for capturing. As detected by scanning electron microscopy (SEM) compared to control beads (top left), NEVs (as indicated by a white arrow) were enriched on the surface of Dynabeads with the mixing effect of herringbone microchannels (Figure 2A). The capturing performance was further verified by introducing Dil-stained NEVs and FAM-labeled CD63 aptamers into the chip. It was observed that Dil-stained NEVs (red) and FAM-labeled aptamers (green) were colocalized and enriched on the surface of Dynabeads (circled with a black dotted line) under a fluorescence microscope, indicating the successful capturing and labeling of NEVs in the chip (Figure 2B).

For better capturing performance, we optimized the microchannel structures and flow rate, which could affect the flow characteristics and fluid mixing in the chip. The herringbone microchannels could change the flow direction and velocity distribution and promote the shear force and disturbance between different fluid layers, thereby disrupting the laminar flow and generating turbulent microvortices, which contributes to the enhanced mixing effect of the fluid flow and highly efficient EV capture.^33,34^ To evaluate the effect of channel structures on the capturing efficiency, we developed chips with herringbone microchannels or straight channels for NEVs capturing. The amount of captured EVs was defined as the difference in protein amount between the original EVs (injected from “inlet 1”) and the recovered EVs (collected at “outlet”), and the capturing efficiency was calculated as the ratio of the captured EVs amount to the original one. As a result, we found that the chip with herringbone microchannels captured significantly higher amount of NEVs and obtained higher capturing efficiency than the chip with straight channels (Figure 2C). In addition, an increased flow rate can enhance the fluid disturbance, but too high a flow rate may induce a short residence time of the fluid, resulting in large flow resistance and insufficient fluid mixing in the flow channel. Herein, we optimized the flow rate from 10 to 0.5 μL/min to further improve the capturing efficiency of the chip. It was found that the amount of captured NEVs increased with the decrease of flow velocity, and more than 90% of NEVs were captured at a flow rate of 0.5 μL/min (Figure 2D). Therefore, a flow rate of 0.5 μL/min was chosen for further use.

Moreover, the capturing specificity of the chip was validated by injecting EVs from other cells, including EVs from lymphocytes (LEVs), human gastric epithelial cells (GES-EVs), and GC cell lines (HGC-EVs and MGC-EVs). The results showed that except for NEVs, all of the other EVs were not captured by the chip, and nearly 90% of them were recovered at “outlet” (EV recovered) (Figure 2E). The above results indicated that the IMCN chip with optimized channel design and flow rate was highly efficient and specific for NEV capturing.

### Verification of the Superiority of IMCN Chip for NEV Separation

To further validate the superiority of the IMCN chip, we compared the quality of NEVs isolated from 20 mL of NCM by ExoQuick, UC, and the chip. For the microfluidic chip, 200 μL of glycine-HCL buffer (pH 2.8) was pumped into the chip to elute NEVs, and the NCM without NEVs was collected at the outlet and set as “Chip waste”. NTA results showed that the average size of NEVs eluted from the microfluidic chip was 138 nm, which was a little bit smaller than those isolated by ExoQuick (169 nm) and UC (154 nm). Moreover, the chip achieved 1.1 × 10^10^ particles/mL of NEVs, while the NEV concentration of ExoQuick and UC was 1.7 × 10^10^ particles/mL and 1.4 × 10^10^ particles/mL, and chip waste obtained the lowest particle concentrations (Figure S6A,B). Although ExoQuick obtained large amounts of nanoparticles, the high particle concentration may be attributed to contamination of lipoproteins. These results were further verified by TEM. As shown in Figure S6C, compared to NEVs isolated by ExoQuick and UC, NEVs of the chip had a complete vesicle structure, uniform particle size, and less contamination, whereas no obvious vesicle particles were observed in chip waste. In addition, NEVs isolated by chip highly expressed CD9, CD63, and HSP70, showing a purity comparable to that of UC (Figure S6D). In contrast, the expression of EV markers was relatively low in the sample of ExoQuick when the same amounts of total proteins were loaded for the Western blot assay. All of the above results verified that NEVs obtained by the IMCN chip had high efficiency and purity (Table S1).

### Optimization of RCA-MB Assay Using Synthetic Sequences

In order to eliminate the tedious EV cargo extraction and detection procedures, we applied on-chip thermal lysis and RCA-MB assay for NEV detection, which enabled nucleic acid amplification and detection under isothermal conditions.^35^ Before the on-chip assay, the feasibility of the RCA-MB assay was verified and optimized in homogeneous solutions, in which the RCA reaction was triggered by synthetic aptamer and miRNAs (Figure S7A; sequences listed in Table S2).

The LPs were prepared as previously reported^36^ and functioned as templates for the RCA reaction. The LPs were obtained by linking the connection probes (CPs) to 5′-phosphorylated padlock probes (PP) and validated by agarose gel electrophoresis. Detailed procedures are described in the Experimental Methods. As shown in Figure S7B, the products exhibited an increased molecular weight of the band (lane 3) after the connection of CP (lane 1) with PP (lane 2) by T4 DNA ligase. Lane 4 showed the same molecular weight of band as lane 2 after exonuclease I and exonuclease III treatment, indicating that the prepared LPs were resistant to enzymatic digestion. Then, we used the synthetic aptamer and miRNAs as primers to trigger RCA reactions, and random sequence primers were used as negative controls (NCs). The amounts of LP, phi29 DNA polymerase, and other reagents were referred to previous studies,^37,38^ and described in detail in the Experimental Methods. For each LP, a bright band was observed only when the specific primers were introduced (lane 5), showing a large molecular weight of the RCA products. On the contrary, no band was found in the NC group. Taken together, these results suggest high specificity of the RCA reaction.

**Figure 3:**
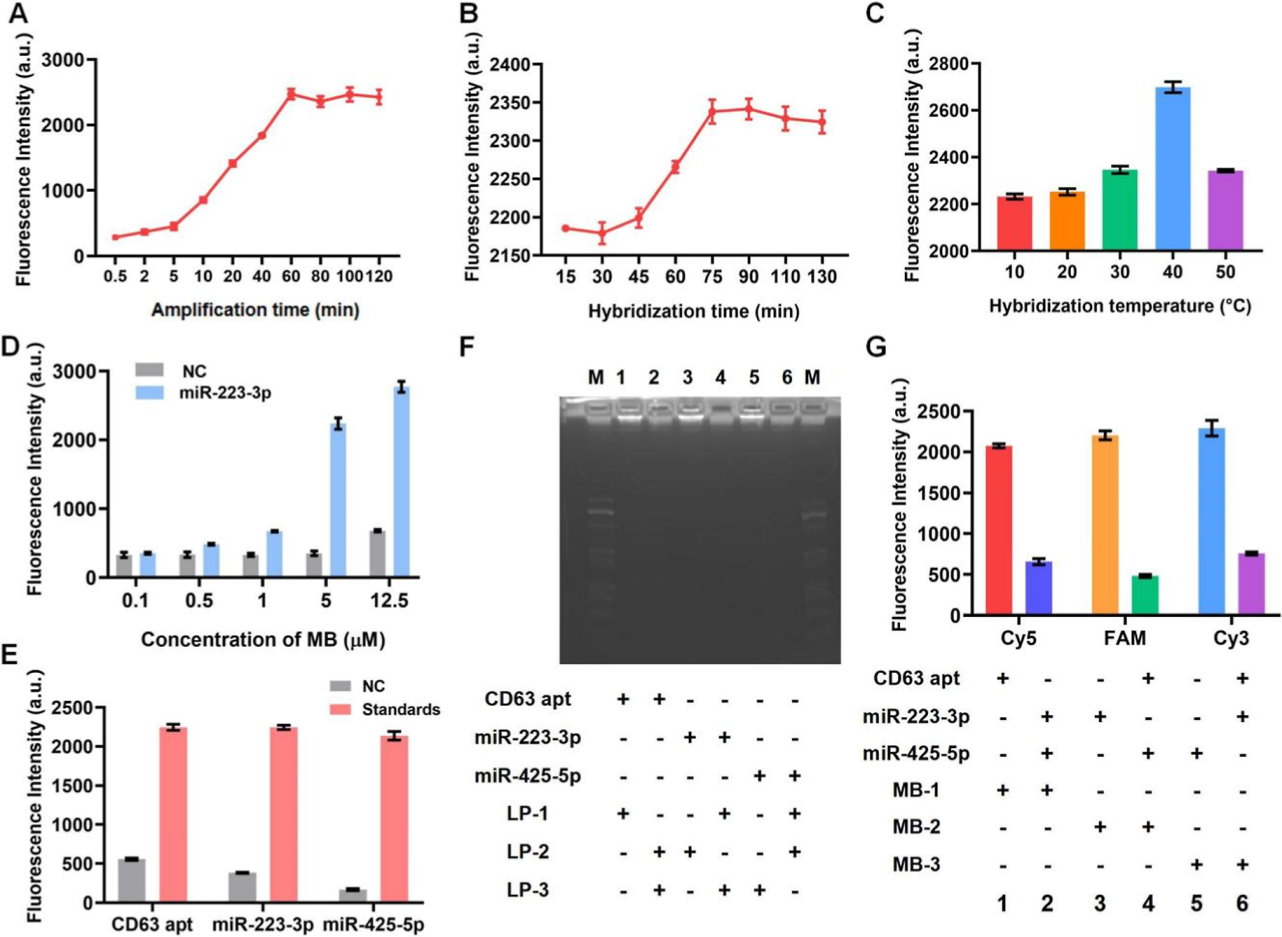
Optimization of the RCA-MB assay using synthetic sequences. (A) Optimization of the amplification time (from 0.5 to 120 min). (B) Optimization of the hybridization time for MBs and RCA products (from 15 to 130 min). (C) Optimization of the hybridization temperature for MBs and RCA products (from 10 to 50 °C). (D) Optimization of the concentration of MBs used in the hybridization reaction (from 0.1 to 12.5 μM). (E) The specificity and feasibility of the triplex RCA-MB assay. (F,G) The specificity of the amplification and detection of the triplex RCA-MB assay. (F) Agarose gel electrophoresis of the RCA products generated by different primers and LPs. (G) Fluorescence intensity of the reaction between different RCA products and MBs.

To obtain the best performance, LP-2, miR-223-3p, and MB-2 were selected to test and optimize the established RCA-MB assay. Figure 3A shows that the RCA reaction tended to reach saturation after amplification for 60 min, and the fluorescence intensity did not increase even with the extension of amplified time. Agarose gel electrophoresis of the RCA products showed the same results (Figure S7C). Moreover, we optimized the time, temperature, and concentration of the hybridization reaction between MBs and the RCA products. It could be observed that the maximum signal-to-noise ratio was obtained when the reaction was performed at 40 °C for 75 min with 5 μM MBs, whereas the background noise greatly increased when MB concentration rose to 12.5 μM (Figure 3B–D). Consequently, the optimized conditions of the RCA-MB assay were used for on-chip NEV detection.

### Detection Performance of RCA-MB Assay Using Synthetic Sequences

We then evaluated the detection performance of the RCA-MB assay by using synthetic aptamers and miRNAs under the optimized conditions. As a result, the fluorescence intensity increased linearly with the increasing concentration of aptamer and miRNAs (ranging from 10^–10^ to 10^–15^ M), and the limit of detection (LOD) was calculated to be 0.77, 0.61, and 0.24 fM for CD63 aptamer, miR-223-3p, and miR-425-5p, respectively (Figure S8A). The LOD was calculated as three times the standard deviation of a set of blank measurements. Moreover, a weak fluorescence signal was detected when MBs were reacted with RCA products triggered by NC, which indicated a good specificity of the assay (Figure S8B). We also used qRT-PCR as the gold standard to measure different concentrations of synthetic miRNAs, and a high correlation coefficient of 0.893 was obtained between the RCA-MB assay and qRT-PCR (Figure S8C,D).

Next, we verified the multiplexed amplification and detection performance of the RCA-MB assay. Three pairs of LPs and their corresponding primers (for CD63 aptamer, miR-223-3p, and miR-425-5p) were added to one tube, and the RCA products were simultaneously reacted with three MBs. We found that the triplex RCA-MB assays obtained similar fluorescent signals to the single assay (Figure 3E). To further verify the specificity of the triplex RCA reaction, the aptamers were reacted with different LPs, and the products were verified by agarose gel electrophoresis. Figure 3F shows that the RCA products were only detected when the aptamers were reacted with LP-1 (lane 1), while no bands were found in the reaction with LP-2 and LP-3 (lane 2). The same results were obtained in miRNAs triggered RCA reactions (lanes 3–6) (Figure 3F). In addition, we used MB-1 to detect the RCA products triggered by different primers (aptamers and miRNAs) to validate the detective specificity of MBs. It was found that the enhanced fluorescence intensity of MB-1 was only detected in aptamer-induced RCA products (histogram 1), while the others did not show significantly enhanced fluorescence signals (histogram 2). The same results were obtained in the detection of miRNAs induced RCA products by MB-2 and MB-3 (histograms 3–6) (Figure 3G). These results indicated that the triplex RCA-MB assay is highly specific in amplification and detection.

To verify the applicability of the RCA-MB assay in NEV detection, we isolated NEVs by UC and incubated them with the aptamers at 37 °C for 1 h and washed with PBS for 5 times to remove unbound aptamers. The aptamer@NEV complex was then heated at 95 °C for 5 min to lyse NEVs and release their derived miRNAs for the multiplexed RCA-MB assay. As shown in Figure S9, the assay could achieve triplex amplification and detection of the CD63 aptamer and two miRNAs from NEVs (miR-223-3p and miR-425-5p). All of the above results indicate that the RCA-MB assay is applicable for simultaneous detection of NEV marker protein and miRNAs.

### Detection of Serum NEV-Derived miRNAs by RCA-MB Assay

To evaluate the potential of the RCA-MB assay in the diagnosis of GC, we collected serum samples from 51 GC patients, 38 BGD patients, and 41 HC. Serum NEVs were isolated as previously reported by our group,^16^ and their derived miRNAs (miR-223-3p and miR-425-5p) were detected by RCA-MB assay. As shown in Figure S10A–D, the fluorescence intensity of miR-223-3p and miR-425-5p was significantly increased in the GC group compared to HC and BGD groups. Moreover, the fluorescence intensity gradually increased with tumor stage, and a higher intensity was observed in I/II stages of the GC group than in the noncancer (HC and BGD) groups (Figure S10E,F). Receiver operating characteristic (ROC) curve indicated that the area under curve (AUC) of miR-223-3p was 0.804 to distinguish GC from HC, and 0.856 to distinguish GC from BGD, while the AUC of miR-425-5p was 0.740 and 0.709, respectively (Figure S10G and Table S3). The combination of two miRNAs showed a higher AUC of 0.853 and 0.872, respectively. Moreover, the AUC of combined miRNAs was 0.811 for I/II stages of GC patients, and 0.937 for III/IV stages of GC patients to differentiate them from noncancer individuals (Figure S10H and Table S4). These results suggest that the RCA-MB assay can efficiently detect miRNAs in serum NEVs and shows a favorable performance in GC diagnosis.

### Detection Performance of RCA-MB-Based IMCN Chip for UC-Isolated NEVs

**Figure 4:**
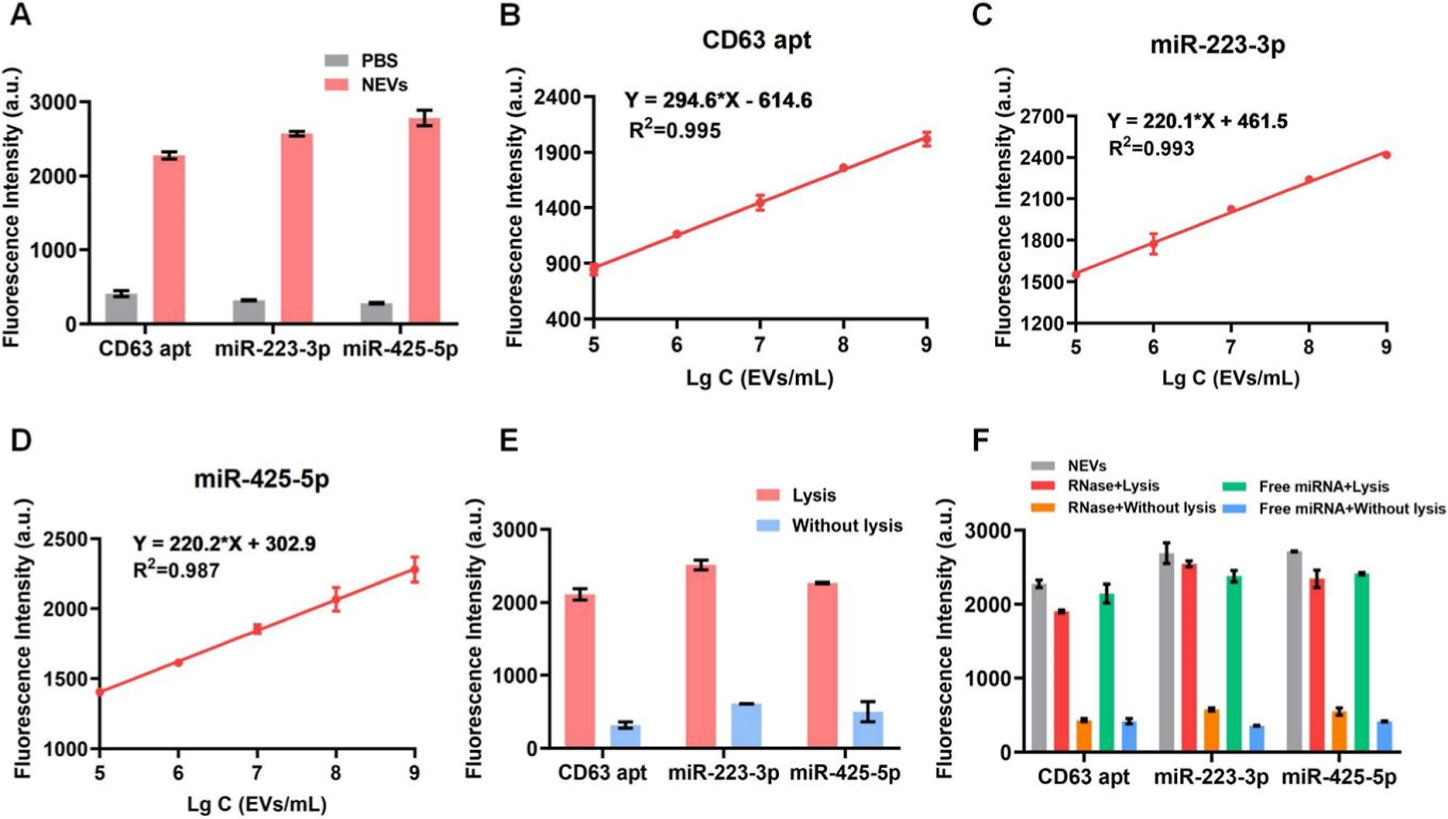
Detection performance of RCA-MB-based IMCN chip using UC-isolated NEVs. (A) Multiplexed analysis and detection of NEVs by the chip. (B–D) Linear range curve of the fluorescence intensities and NEVs with gradient concentrations (B) CD63 aptamers, (C) miR-223-3p, and (D) miR-425-5p. (E) Verification of the specificity of the fluorescence signals from NEV samples as detected by the chip. (F) Anti-interference performance of the chip in the presence of high concentrations of free miRNAs and RNase.

The RCA-MB assay was then applied on the IMCN chip to evaluate its detection performance by using UC-isolated NEVs. As shown in Figure 4A, significant fluorescence signals were obtained by on-chip analysis of NEVs, and the expression of CD63 aptamers, miR-223-3p and miR-425-5p, was consistent with that measured by off-chip examination. Next, NEVs with gradient concentrations were used to assess the detective sensitivity. The fluorescence intensities were found to be increased with the increase of NEV concentrations, and a good linear correlation was obtained when the concentrations of NEVs ranged from 10^5^ to 10^9^ EVs/mL (Figure 4B–4D). The detection limit for CD63 aptamers, miR-223-3p and miR-425-5p, was estimated to be 19.5 × 10^4^, 2.2 × 10^4^ and 1.6 × 10^4^ EVs/mL, respectively. For specificity validation, Figure 4E shows that enhanced fluorescence signals were only observed when the NEVs were lysed on the chip, while the signals were weak without NEV lysis, indicating that the fluorescence signals specifically originated from the lysed NEVs. To further validate the anti-interference ability of the IMCN chip, UC-isolated NEVs were mixed with high concentrations of RNase (2 U, 20 U/μL) or free miRNAs (10 μM) to simulate the complicated components of human serum samples, and they were then applied on the chip. As can be seen in Figure 4F, no enhanced fluorescence signal was detected when NEVs were not lysed (histograms in yellow and blue), even with the addition of a high concentration of RNase or free miRNAs. This may be attributed to the complete removal of impurities by channel washing and the protective effect of miRNA cargos by the complete double-layer vesicle structure of NEVs. In contrast, significantly enhanced fluorescence signals can only be observed after NEV lysis (histogram in red and green), indicating that the detected signals were specifically from NEVs. Therefore, the NEV detection by the IMCN chip was stable in the presence of high concentrations of RNase, and the addition of high concentrations of free miRNAs did not cause unspecific signals. In summary, the IMCN chip has good sensitivity and specificity to detect NEVs and low interference by nuclease and free nucleic acid in serum samples.

For repeatability evaluation, UC-isolated NEVs were applied on the chip at a concentration of 10^9^ EVs/mL. The amounts of CD63 aptamers and expression levels of miRNAs were continuously measured for 4 days (three times per day). Relative standard deviation (RSD) was used to describe the interday and intraday accuracy of the chip. As a result, no statistically significant difference was observed between the measurements (Figure S11). For the amount of CD63 aptamers, the intraday RSD was 1.54%, and the interday RSD was 2.59%. For miR-223-3p and miR-425-5p expression, the intraday and interday RSD were 0.79% and 0.65% and 1.37% and 1.91%, respectively. These results suggest that the RCA-MB assay-based IMCN chip has great stability and repeatability.

### Detection of Human Serum Samples by IMCN Chip and Evaluation of Its Diagnostic Performance

**Figure 5:**
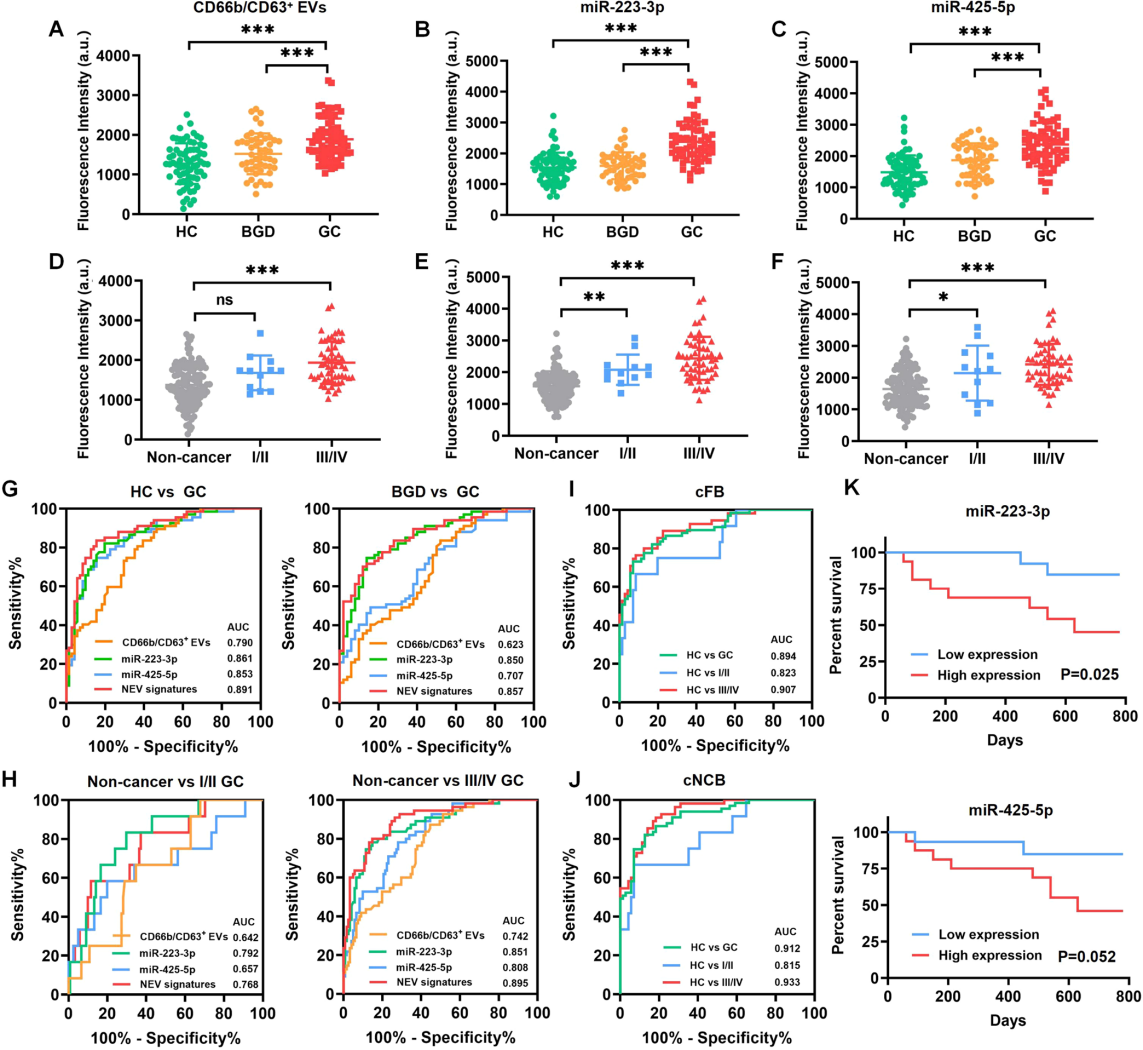
Detection of human serum samples by IMCN chip and evaluation of its diagnostic performance. (A–C) The amount of (A) CD66b/CD63^+^ NEVs and expression of (B) miR-223-3p and (C) miR-425-5p in HC, BGD, and GC groups (***P < 0.001). (D–F) The amount of (D) CD66b/CD63^+^ NEVs and expression of (E) miR-223-3p and (F) miR-425-5p in HC and GC of different stages (***P < 0.001). (G) ROC curves of NEV signatures in distinguishing between HC and GC, BGD and GC groups. (H) ROC curve of NEV signatures in distinguishing between noncancer and GC of I/II and III/IV stages. (I) ROC curve of the combination of four used biomarkers, CD66b/CD63^+^ NEVs, miR-223-3p, CEA, and CA199 (cFB). (J) ROC curve of the combination of NEV signatures and commonly used biomarkers (cNCB). (K) The prognostic value of miR-223-3p and miR-425-5p (as detected in NEVs by the chip) in GC patients.

We next performed on-chip isolation of NEVs and detection of NEV signatures by introducing 10 μL of serum samples from different individuals. The diagnostic performance of the IMCN chip was evaluated by using a cohort containing 71 HC, 50 BGD patients, and 67 GC patients. As shown in Figure 5A–C, the amount of CD66b/CD63^+^ NEVs (as indicated by CD63 aptamer fluorescence signal) and the expression levels of NEV-derived miRNAs were significantly increased in the GC group compared to the HC and BGD groups. The fluorescence signals varied in GC patients with different stages, and a higher intensity was obtained in I/II stages of GC patients than in noncancer (HC and BGD) individuals (Figure 5D–F). The ROC curve indicated that the amount of CD66b/CD63^+^ NEVs and the expression levels of NEV-derived miRNAs exhibited good performance in GC diagnosis. As shown in Figure 5G, the AUC value of CD66b/CD63^+^ NEVs to differentiate between HC and GC was 0.790, while miR-223-3p and miR-425-5p were 0.861 and 0.853, respectively. Moreover, the combination of three NEV biomarkers (NEV signatures) showed better differentiation of HC and GC, with an AUC of 0.891 (83.58% of sensitivity and 84.51% of specificity). NEV signatures could well distinguish between GC and BGD groups, with an AUC of 0.857 (70.15% sensitivity and 88.00% specificity; Figure 5G). In addition, when comparing GC patients of different stages with noncancer individuals (BGD and HC), NEV signatures showed a higher diagnostic accuracy than a single biomarker, with an AUC of 0.768 for GC in I/II stages and 0.895 for GC in III/IV stages (Figure 5H).

In addition, we investigated the diagnostic performance of CEA and CA199 in the same cohort. The ROC curve showed that the AUC value of CEA and CA199 was 0.618 and 0.682 to distinguish GC patients from HC, while an AUC of 0.704 was achieved when these two common biomarkers (cCB) were combined (Figure S12A,B). Similarly, the combination of CEA and CA199 did not behave as good as NEV signatures to differentiate between GC of different stages and HC, with an AUC of 0.707 for GC in I/II stages and 0.712 for GC in III/IV stages (Figure S12C,D).

We further analyzed the diagnostic performance of NEV signatures combined with the commonly used biomarkers. The ROC curve indicated that the diagnostic value was markedly improved by combing four biomarkers (CD66b/CD63^+^ NEVs, miR-223-2p, CEA, and CA199, termed as cFB), with an AUC of 0.894 to differentiate GC patients from HC, 0.823 for GC in I/II stages, and 0.907 for GC in III/IV stages (Figure 5I). Moreover, the AUC value was further increased to 0.912, 0.815, and 0.933 when miR-425-5p was added (cNCB) (Figure 5J). In addition, we found that the high levels of miR-223-3p in NEVs predicted poor prognosis in GC patients, while miR-425-5p, CEA, and CA199 did not show significant correlation with the patients’ survival (Figures 5K and S12E,F). Hence, NEV signatures showed superior diagnostic performance to the conventional biomarkers, and their combinations further improved GC diagnosis (Tables S5–S7).

### Diagnostic Performance of IMCN Chip Assisted with Machine Learning for Gastric Cancer Diagnosis

**Figure 6:**
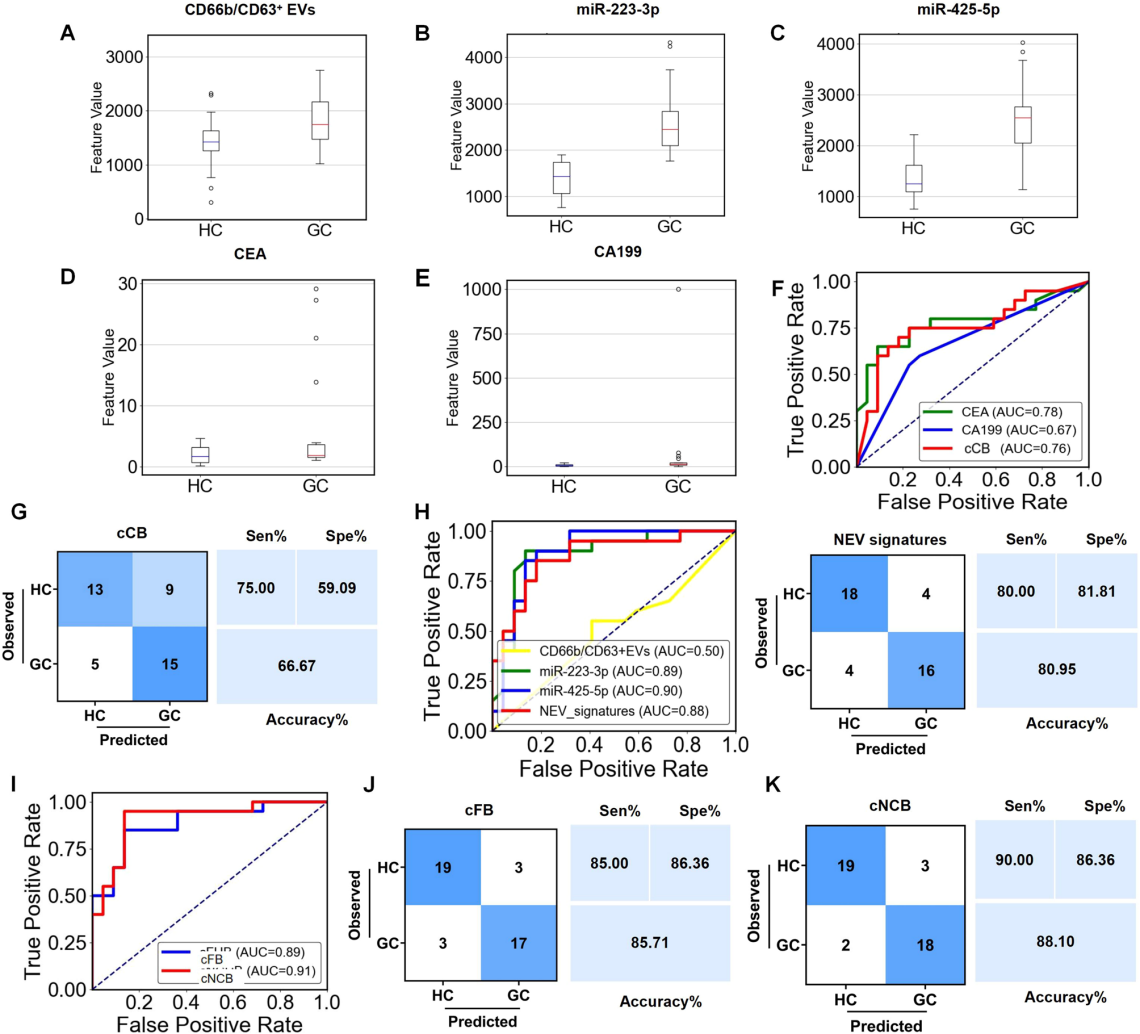
Diagnostic performance of IMCN chip assisted with machine learning for GC diagnosis. (A–E) The predicted value of different biomarkers to distinguish between HC and GC under the RF model. (F–G) ROC curves and the confusion matrix of CEA, CA199, and their combinations (cCB) to distinguish GC from HC. (H) ROC curves and confusion matrix of NEV signatures to distinguish GC from HC. (I) ROC curves of the combination of four used biomarkers, CD66b/CD63^+^ NEVs, miR-223-3p, CEA, and CA199 (cFB), and the combination of NEV signatures, CEA, and CA199 (cNCB) to distinguish GC from HC. (J) The confusion matrix of cFB to distinguish between HC and GC. (K) The confusion matrix of cNCB to distinguish between HC and GC.

RF is a randomly constructed classifier that contains multiple decision trees and is commonly used to accomplish prediction tasks.^39^ To further improve the performance of the IMCN chip in GC diagnosis, 70% of samples (48 GC and 49 HC) was randomly selected and trained to construct a diagnostic model by RF on the combined biomarkers (NEV signatures, CEA, and CA199), and 30% (20 GC and 22 HC) was used for testing. The predicted value of different biomarkers to distinguish between HC and GC under the RF model is presented in Figure 6A–E. ROC analysis was then performed to determine the sensitivity, specificity, and AUC value of a single marker and the combined biomarkers. The performance of CEA and CA199 was improved under RF (Figure 6F), showing an AUC value of 0.78 for CEA and 0.67 for CA199, and 0.76 for the two combined (cCB, with 75.00% sensitivity and 59.09% specificity; Figure 6G). The diagnostic efficiency of NEV signatures was relatively high, showing an AUC value of 0.88 (with 80.00% sensitivity and 81.81% specificity; Figure 6H). In addition, the diagnostic performance gradually increased when more biomarkers were used. The combination of four biomarkers (CD66b/CD63^+^ NEVs, miR-223-2p, CEA, and CA199, termed cFB) yielded an AUC of 0.89 (with 85.00% sensitivity and 86.36% specificity) (Figure 6I,J). The diagnostic value of NEV signatures combined with commonly used biomarkers (cNCB) increased significantly in the RF-assisted diagnostic model (AUC of 0.91, 90.00% of sensitivity, and 86.36% of specificity) (Figure 6I,K). These results indicate that the diagnostic performance of NEV signatures as detected by the IMCN chip for GC diagnosis was significantly improved when combined with conventional biomarkers with the assistance of machine learning (Tables S8 and S9).

## Conclusions

In summary, we present here an IMCN analysis and GC diagnosis. CD66b antibody-coupled Dynabeads incorporating CD63 aptamers enabled rapid and efficient separation of CD66b/CD63^+^ NEVs, which avoids the lengthy isolation process of UC and lipoprotein contamination by ExoQuick methods.^40^ Optimized herringbone microchannel and flow rate facilitated more than 90% capturing of NEVs, which is higher than that reported in our previous study and other studies.^16,24^ After NEVs enrichment, all of the wastes and potential contaminants in serum could be thoroughly cleared by chip channel washing, thereby reducing the unspecific background noise. In addition, the membranous vesicle structure of NEVs could effectively resist the degradation by nucleases in the biofluids and well protect the carried miRNAs to be measured, which facilitated specific NEV analysis and increased detection accuracy.^41^ Moreover, on-chip thermal lysis and RCA-MB assays were established and optimized, which favored simultaneous and triplex detection of CD66b/CD63^+^ NEVs and their contained miRNAs (NEV signatures). The method endows simple and low-cost nucleic acid amplification and detection under isothermal conditions,^42−44^ which is free from the process of cargo extraction and avoids the expensive reagents and special instruments required by the previously reported studies^28,39,45^ (Table S12).

The IMCN chip is confirmed with high sensitivity, specificity, and repeatability. By using only 10 μL of serum samples, the chip achieved efficient and automatic detection of NEV signatures, which showed good performance in distinguishing among HC, BGD, and GC patients, as well as GC of different stages. In addition, the diagnostic accuracy of NEV signatures was significantly higher than that of commonly used serum biomarkers, and their combination achieved a higher diagnostic accuracy. Machine learning has shown great potential in cancer precision diagnosis.^46,47^ We further used the RF algorithm to analyze the readout of the NEV signatures. The diagnostic performance of the NEVs was significantly improved under the RF model, and the highest diagnostic accuracy was obtained by the five combined biomarkers (NEV signatures and CEA, CA199).

In the future, we will further conduct a multicenter clinical trial to validate the performance of the IMCN chip. We will optimize chip fabrication to reduce production costs, increase stability and reliability, and improve analytic performance. In addition, we will design a chip for the high-throughput NEV analysis of multiple samples. Moreover, the development of automatic sampling equipment will reduce manual operations and enhance detection accuracy and reproducibility.

## Experimental Methods

### Study Design

The objective of this study was to develop an IMCN for isolation and multiplexed detection of NEV-derived miRNAs. For NEV separation, a 3D herringbone micromixer was constructed and optimized to promote rapid, efficient, and high-purity enrichment of NEVs by using CD66b antibody-coupled Dynabeads. CD63 aptamers were introduced to recognize NEVs and detect the amount of CD66b/CD63^+^ NEVs. The RCA-MB assay was established for nucleic acid amplification and signal detection under isothermal conditions. The assay was optimized and evaluated in off-chip reactions and then applied to the chip. The fluorescence signal was measured via a cell imaging microplate detection system (Cytation 5, BioTek) at an excitation wavelength of 484 nm for FAM, 540 nm for Cy3, and 640 nm for Cy5. The measurement was repeated three times for each sample and each experiment. Serum samples of HC, BGD, and GC groups (aged ranging from 50 to 90) were collected and tested by the chip. The diagnostic performance of the NEV signatures and commonly used biomarkers was evaluated in the diagnosis, staging, and prognosis of GC and was further analyzed by a machine learning-based ensemble classification system with single and combined biomarkers.

## Materials and Reagents

Injection pump was bought from Longer (LSP02-2A; Shanghai, China). Fetal bovine serum (FBS) and RPMI 1640 were purchased from Gibco (NY, USA). ExoQuick-TC (EXOTC10A-1) was purchased from System Biosciences (CA, USA). 100 kDa ultrafiltration centrifugal tubes were purchased from Millipore (MA, USA). Dynabeads antibody coupling kit, Dil cell membrane staining reagent, and aldehyde/sulfate latex beads were acquired from Invitrogen (CA, USA). Phosphate buffer solution (PBS, pH 7.3–7.5) was purchased from Servicebio (Wuhan, China). Yeast RNA was purchased from Beyotime (Shanghai, China). Gold-labeled goat antirabbit IgG antibody was obtained from Wokai (Beijing, China). Anticalnexin antibody (ab75801), anti-CD63 antibody (ab271286), and anti-CD66b antibody (ab300122) were purchased from Abcam (Cambridge, UK). Anti-CD9 antibody (13404) and anti-HSP70 antibody (4873) were purchased from Cell Signaling Technology (MA, USA). BCA protein quantification kit, MiRNA first strand cDNA synthesis kit, and AceQ qPCR SYBR Green Master Mix were obtained from Vazyme (Nanjing, China). MiRNeasy serum/plasma kit was obtained from QIAGEN (Dusseldorf, Germany). DNA marker, aptamers, and all the nucleic acid sequences were provided by Sangon (Shanghai, China) (Table S2). T4 DNA Ligase, exonuclease I, and exonuclease III were acquired from New England Biolabs (MA, USA). Phi29 DNA polymerase and dNTP Mix were purchased from CWBIO (Beijing, China).

### Cell Culture and EV Isolation

#### Neutrophils Isolation and Culture

Neutrophils were isolated from the peripheral blood of healthy volunteers by polymorphprep (Axis-Shield Po CAS, Norway) and cultured in RPMI 1640 supplemented with 10% EVs-free FBS at the conditions of 5% CO_2_ and 37 °C for 24 h.

#### NEV Isolation by UC and ExoQuick

The NCM was collected and sequentially centrifuged at 300*g* for 20 min, 2000*g* for 20 min, and 10,000*g* for 30 min. The supernatants were then filtered through 0.22 μm Millex-GV filter and concentrated by 100 kDa ultrafiltration centrifugal tubes. After that, the NCM was ultracentrifuged at 100,000*g* for 70 min twice, and the NEV pellets were resuspended in PBS and stored at −80 °C until use. For ExoQuick, the reagents were added to the NCM at a ratio of 1:5 and precipitated at 4 °C overnight. On the next day, the NEV precipitates were obtained by centrifugation at 1500*g* for 30 min and redissolved in PBS.

### NEVs Characterization

#### Nanoparticle Tracking Analysis

For NTA (ZetaView, Particle Metrix), the NEV samples were diluted to 1 mL and injected into the NTA chamber. The samples were measured in scatter mode with a 488 nm laser for size distribution and concentration measurement.

#### Western Blot

NEVs were lysed by radio immunoprecipitation assay (RIPA) lysis buffer containing a protease inhibitor cocktail, and the protein concentration was determined by the BCA protein quantification kit. Next, equal amounts of NEVs obtained by different methods were separated by SDS-polyacrylamide gel electrophoresis and then transferred onto the polyvinylidene difluoride (PVDF) membrane. After blocking with 5% bovine serum albumin (BSA) for 2 h, the membrane was incubated with primary antibodies at 4 °C overnight. The next day, the membrane was incubated with horseradish peroxidase (HRP)-conjugated secondary antibodies at room temperature for 2 h, and the band signals were detected by the enhanced chemiluminescence reagent (Beyotime, Beijing, China). NEVs were quantified as protein amounts by the BCA Protein Assay Kit, and the particle concentrations were measured via NTA.

#### Transmission Electron Microscopy

The morphology of the NEVs was characterized by TEM (HT7800, Hitachi). For immune electron microscopy, 20 μL of NEVs was fixed on copper grids for 5 min at room temperature. Next, the NEV samples were sequentially incubated with anti-CD66b primary antibody and gold-labeled secondary antibody (10 nm gold nanoparticles) for 30 min. After that, 20 μL of 2% phosphotungstic acid was added to stain for another 5 min, and the excess solution was washed away with PBS. Finally, the samples were dried for TEM observation.

### NEVs Recognition by Aptamers

#### Fluorescence Microscope

Aptamers were heated to 95 °C and slowly cooled to room temperature before use. NEVs were loaded on polylysine-coated slides and fixed at room temperature for 30 min. Next, 2 μL of Dil membrane staining reagent with 50 μL of BSA/PBS (2%) was added and stained for another 30 min. The slides were then washed with PBS, followed by incubation with 1 μM FAM-labeled CD63 aptamer for 1 h. After washing twice, the slides were blocked and examined under a laser confocal fluorescence microscope (GE, USA).

#### Immunofluorescence

NEVs were fixed on the poly-l-lysine-coated slides at room temperature for 30 min and incubated with the primary antibodies against CD66b (1:50, rabbit antihuman) and CD63 (1:50, mouse antihuman) at 4 °C overnight. The slides were washed with PBS twice to remove the unbound antibodies, followed by incubation with Cy3-labeled secondary antibodies (rabbit) and FITC-labeled secondary antibodies (mouse) for 1 h. After three washings with PBS, the slides were blocked with neutral resin and examined under a laser confocal fluorescence microscope.

#### Flow Cytometry

NEVs were attached to aldehyde/sulfate latex beads by mixing 10 μL EVs with 10 μL latex beads (4% w/v) for 15 min at room temperature with continuous rotation.^48^ The mixture was then diluted to 1 mL by PBS and continually rotated for 30 min. The reaction was stopped with 100 μL of 1 M glycine and 20% BSA/PBS and left 2 h rotating at room temperature. After that, the NEV-bound beads were washed with 2% BSA/PBS at 10,000 rpm for 1 min, blocked with 10% BSA with rotation at room temperature for 30 min, and finally resuspended in 50 μL 2% BSA/PBS. Afterward, 1 μL of NEV-bound latex beads was diluted 50 times and incubated with 1 μM FAM-labeled CD63 aptamer in 450 μL of aptamer binding buffer (4.5 g/L glucose, 5 mM MgCl_2_, 1 mg/mL BSA, and 0.1 mg/mL yeast tRNA) during 2 h rotating at 37 °C. After that, the mixture was washed two times with aptamer washing buffer (4.5 g/L glucose and 5 mM MgCl_2_) for 1 min at 3000*g*. The NEV-bound latex beads were finally dissolved in 400 μL of PBS for flow cytometry analysis (CytoFLEX, Beckman).

## Preparation of CD66b Antibody-Coupled Dynabeads

The preparation and characterization of CD66b antibody-coupled Dynabeads was previously described.^16^ Briefly, 12 μg of anti-CD66b antibody and 2 mg of Dynabeads were conjugated through covalently binding by reaction at 37 °C for more than 18 h. After sequential washing, the antibody-coupled Dynabeads were finally resuspended in PBS at a final concentration of 0.1 mg/mL for further use.

### Design and Preparation of the Microfluidic Chip

#### Design and Fluid Simulation of the Microfluidic Chip

The IMCN chip is 85 mm × 30 mm in size and has five holes (3 mm in diameter), four sets of herringbone mixer channels, a magnetic capture chamber, and a reaction chamber (10 mm in diameter). Each mixer channel had four groups of asymmetrical herringbone grooves, and each group contained 20 grooves with a spacing of 0.1 mm. Fluid dynamics in the chip were analyzed by performing velocity and pressure simulations through Multiphysics 5.2 software (COMSOL, MA, USA). The simulation was based on realizable k-ε turbulence model and Navier–Stokes’ equation for three-dimensional incompressible fluid. The flow velocity at the inlet was set at 2.4 mm/s. The boundary conditions were set to a velocity inlet and a pressure outlet of 1 atm. Different channel aspect ratios were set in the simulation (80:40, 80:50, 100:40, and 100:50 μm), and the streamline charts and pressure profile were obtained to evaluate the mixing effect.

#### Microfluidic Chip Fabrication

Soft lithography was applied for microfluidic chip fabrication. Briefly, the flow channel template was prepared by a lithographic machine and SU8 negative photoresist according to CAD drawings. The PDMS prepolymer and curing agent were stirred at a mass ratio of 10:1 and slowly covered the flow channel template after bubble removal by vacuum kettle. After being baked and solidified, the PDMS chip was carefully removed and then cut and punched as designed. Finally, the chip was put into a plasma machine for plasma treatment and then baked in an oven again to improve the bonding quality.

#### Scanning Electron Microscopy

The prepared Dynabeads and NEV suspensions were driven into the chip for NEV capture and enrichment. After continuous phosphate balanced solution (PBS) and air washing, the chip was kept as dry as possible. Then the external magnetic field was removed, and the conductive material was attached to the bottom of the chip. Finally, the chip was placed on the sample table and vacuumed for 30 min, and the NEVs captured by Dynabeads (NEVs@Dynabeads) were observed under SEM with backscatter mode (Phenom XL).

### RCA Reaction Assay and MB Detection

#### Preparation of Loop Probes

To prepare LP, 6 μL of the PP (10 μM) was hybridized with 6 μL of 10 μM CP in 5 μL of 10 × T4 DNA ligase reaction buffer at 85 °C for 5 min and slowly cooled to room temperature. Next, the hybridization product was added to a ligation mixture containing 1 μL of T4 DNA ligase (10U/μL) and 32 μL of DEPC water. The ligation process was performed at 25 °C for 3 h and stopped at 65 °C for 10 min. Then, 2 μL of exonuclease I (20U/μL) and 2 μL of exonuclease III (100U/μL) were added into the ligation product at 37 °C overnight to eliminate the unlooped linear nucleic acids.

#### RCA Reaction

The RCA reaction was carried out in a mixture containing 1 μL LP, 2 μL 10× phi29 DNA polymerase reaction buffer, 2 μL dNTPs (10 mM for each of dATP, dGTP, dCTP, and dTTP), 1 μL phi29 DNA polymerase (10U/μL), synthesized miRNAs or aptamers (or lysed NEVs), and DEPC water (20 μL in total). The mixture was incubated at 37 °C for 1 h and heated at 65 °C for 15 min to stop the reaction. The random sequence was used as the NC. The synthesis of LP and the products of the RCA reaction were verified by agarose gel electrophoresis.

#### Agarose Gel Electrophoresis

Microwaving a triangular bottle with 30 mL of Tris-Borate-EDTA (TBE) buffer and 0.9 g of agarose to boil. The gel was cooled to 60 °C and then mixed with a nucleic acid dye. The samples were loaded after the gel solidified, and the electrophoresis was carried out at 120 V under a constant voltage. The gel was finally observed and photographed by a UV analyzer.

#### Detection of RCA Products by MBs

Ten μL of RCA products was mixed with different concentrations of MBs and diluted to a final volume of 30 μL by DEPC water. The mixture was incubated at different temperatures or for different durations. The fluorescence signal was measured via a cell imaging microplate detection system (Cytation 5, BioTek) at an excitation wavelength of 484 nm for FAM, 540 nm for Cy3, and 640 nm for Cy5.

#### On-Chip RCA Reaction and MB Detection

A mixture containing 3 μL three kinds of LP, 2 μL 10× phi29 DNA polymerase reaction buffer, 2 μL dNTPs (10 mM for each of dATP, dGTP, dCTP, and dTTP), and 1 μL phi29 DNA polymerase was driven and incubated with the lysed NEVs. On-chip RCA reaction was performed on an electronic hot plate (KAISI) at 37 °C for 1 h and stopped at 65 °C for 15 min. Next, 3 μL of a mixture of three MBs was injected through “inlet 4” for NEV quantification and miRNA detection. After incubation for 1 h, the air was pumped into the channels to ensure complete collection of liquids from the channel, and the fluorescence intensity of the MBs was measured by a cell imaging microplate detection system (Cytation 5, BioTek).

## Patients and Clinical Samples

The clinical samples of GC patients, BGD patients, and HC were obtained from the Affiliated People’s Hospital of Jiangsu University. This study was approved by the Ethics Committee of Jiangsu University (UJS-IACUC-AP-2023022703) and was conducted in accordance with the ethical principles of the World Medical Association Declaration of Helsinki. Informed consent was obtained from all of the subjects. GC patients were included with histology confirmation and primary diagnosis without surgical treatment, radiotherapy, or chemotherapy. The patients with multiple primary cancers were excluded. Before use, the serum samples were centrifuged at 1500*g* for 15 min and 10000*g* for 20 min to remove cell debris and large vesicles.

For off-chip detection, ultracentrifuged and Dynabeads-separated serum NEVs were lysed by heating the NEVs@Dynabeads in 10 μL of DEPC water at 95 °C for 5 min. The lysis solution was then adapted for the RCA reaction and MB detection in a homogeneous solution as described above. For on-chip serum NEV detection, 10 μL of diluted serum samples (1:10 in PBS) was injected to the chip together with aptamers and antibody-coupled Dynabeads. All of the other steps were the same as described above. The information on clinical samples used in RCA-MB assay and on-chip NEV analysis is listed in Tables S10 and S11, respectively.

### Development of a Machine Learning-Assisted Diagnostic Model

The data of five biomarkers (NEV signatures, CEA, and CA199) was analyzed by a machine learning model (RF) developed via Python 3.9.0. Each random decision tree of the model performed binary classifications between HC and GC cases, with the final outcome determined by a majority vote across all biomarker panels. For model construction, a hyperparameter tuning process was employed for optimal algorithm performance. The RandomizedSearchCV function was utilized to explore potential hyperparameters to identify the best set of parameters that maximized the model’ s accuracy while minimizing overfitting. The model was trained by a supervised learning method, and the enrolled patients were randomly divided into 70% training sets and 30% independent testing sets to validate the diagnostic performance. The expression of five biomarkers from testing set samples was analyzed by the model, and the generated predicted values of single biomarker and their combinations were further analyzed by ROC curves to evaluate the diagnostic efficiency. A confusion matrix was applied to determine the diagnostic sensitivity, specificity, and accuracy. The source code is available from https://github.com/yudan911/RF-model/blob/main/main2.py.

### Statistical Analysis

All experiments were performed in triplicate of each group, and GraphPad Prism (PRISM version 9) software was used to construct bar plots, correlation analysis, ROC curve analysis, and PCA analysis. Shapiro–Wilk test and the Kolmogorov–Smirnov test were used to verify the normality of the data, and the Levene test was applied to verify the homogeneity of the variance. The statistical significance of the difference was performed by Student’ s *t* test if data were normally distributed. Otherwise, one-way analysis of variance (ANOVA) and two-way ANOVA for multiple groups were determined. If the data did not meet the assumptions of these parameter tests, nonparametric testing methods or data transformation were then performed. Survival time was analyzed by the Kaplan–Meier method and a log-rank test. The correlation between the outcome of the RCA-MB assay and qRT-PCR was evaluated by Pearson correlation coefficients. *P* < 0.05 was considered significant. The 95% CIs for sensitivity and specificity in GC diagnosis and staging were estimated by the Clopper–Pearson method.

## Data Availability

All data are available in the main text or the Supporting Information.

## Supporting Information Available

Details on additional materials including the design principle and images of developed IMCN chip, characterization of UC-isolated NEVs, characterization of aptamer binding activity and stability, characterization of NEVs separated by different methods, characterization and performance evaluation of RCA-MB assay, detection repeatability of the chip, nucleic acid sequences, clinical information, diagnostic performance of different biomarkers, and comparison of previous studies and this work (PDF)

## References

[ref1] Sung H.; Ferlay J.; Siegel R. L.; Laversanne M.; Soerjomataram I.; Jemal A.; Bray F. Global Cancer Statistics 2020: GLOBOCAN Estimates of Incidence and Mortality Worldwide for 36 Cancers in 185 Countries. Ca-Cancer J. Clin. 2021, 71 (3), 209–249. 10.3322/caac.21660. 33538338

[ref2] Arya S. B.; Collie S. P.; Parent C. A. The Ins-And-Outs of Exosome Biogenesis, Secretion, and Internalization. Trends Cell Biol. 2024, 34 (2), 90–108. 10.1016/j.tcb.2023.06.006. 37507251 PMC10811273

[ref3] Li Y.; Sui S.; Goel A. Extracellular Vesicles Associated MicroRNAs: Their Biology and Clinical Significance as Biomarkers in Gastrointestinal Cancers. Semin. Cancer Biol. 2024, 99, 5–23. 10.1016/j.semcancer.2024.02.001. 38341121 PMC11774199

[ref4] Lei Y.; Fei X.; Ding Y.; Zhang J.; Zhang G.; Dong L.; Song J.; Zhuo Y.; Xue W.; Zhang P.; Yang C. Simultaneous Subset Tracing and MiRNA Profiling of Tumor-Derived Exosomes via Dual-Surface-Protein Orthogonal Barcoding. Sci. Adv. 2023, 9 (40), eadi1556 10.1126/sciadv.adi1556. 37792944 PMC10550235

[ref5] Zhang Y.; Wu Q.; Huang Y.; Wang W.; Lu Y.; Kang S.; Yang C.; Song Y. Reliable Detection of Extracellular PD-L1 by DNA Computation-Mediated Microfluidics. Anal. Chem. 2023, 95 (24), 9373–9379. 10.1021/acs.analchem.3c01686. 37276048

[ref6] Zhang C.; Qin C.; Dewanjee S.; Bhattacharya H.; Chakraborty P.; Jha N. K.; Gangopadhyay M.; Jha S. K.; Liu Q. Tumor-Derived Small Extracellular Vesicles in Cancer Invasion and Metastasis: Molecular Mechanisms, and Clinical Significance. Mol. Cancer 2024, 23 (1), 18 10.1186/s12943-024-01932-0. 38243280 PMC10797874

[ref7] Kang Y.-T.; Niu Z.; Hadlock T.; Purcell E.; Lo T.-W.; Zeinali M.; Owen S.; Keshamouni V. G.; Reddy R.; Ramnath N.; Nagrath S. On-Chip Biogenesis of Circulating NK Cell-Derived Exosomes in Non-Small Cell Lung Cancer Exhibits Antitumoral Activity. Adv. Sci. 2021, 8 (6), 2003747 10.1002/advs.202003747. PMC7967048 33747745

[ref8] Hong J.-S.; Son T.; Castro C. M.; Im H. CRISPR/Cas13a-Based MicroRNA Detection in Tumor-Derived Extracellular Vesicles. Adv. Sci. 2023, 10 (24), e2301766 10.1002/advs.202301766. PMC10460892 37340600

[ref9] Hedrick C. C.; Malanchi I. Neutrophils in Cancer: Heterogeneous and Multifaceted. Nat. Rev. Immunol. 2022, 22 (3), 173–187. 10.1038/s41577-021-00571-6. 34230649

[ref10] Tian S.; Chu Y.; Hu J.; Ding X.; Liu Z.; Fu D.; Yuan Y.; Deng Y.; Wang G.; Wang L.; Wang Z. Tumour-Associated Neutrophils Secrete AGR2 to Promote Colorectal Cancer Metastasis via Its Receptor CD98hc-xCT. Gut 2022, 71 (12), 2489–2501. 10.1136/gutjnl-2021-325137. 35086885

[ref11] Wang J.; Wang X.; Guo Y.; Ye L.; Li D.; Hu A.; Cai S.; Yuan B.; Jin S.; Zhou Y.; Li Q.; Zheng L.; Tong Q. Therapeutic Targeting of SPIB/SPI1-Facilitated Interplay of Cancer Cells and Neutrophils Inhibits Aerobic Glycolysis and Cancer Progression. Clin. Transl. Med. 2021, 11 (11), e588 10.1002/ctm2.588. 34841706 PMC8567044

[ref12] Tyagi A.; Wu S.-Y.; Sharma S.; Wu K.; Zhao D.; Deshpande R.; Singh R.; Li W.; Topaloglu U.; Ruiz J.; Watabe K. Exosomal MiR-4466 from Micotine-Activated Neutrophils Promotes Tumor Cell Stemness and Metabolism in Lung Cancer Metastasis. Oncogene 2022, 41 (22), 3079–3092. 10.1038/s41388-022-02322-w. 35461327 PMC9135627

[ref13] Mattox A. K.; Douville C.; Wang Y.; Popoli M.; Ptak J.; Silliman N.; Dobbyn L.; Schaefer J.; Lu S.; Pearlman A. H.; Cohen J. D.; Tie J.; Gibbs P.; Lahouel K.; Bettegowda C.; Hruban R. H.; Tomasetti C.; Jiang P.; Chan K. C. A.; Lo Y. M. D.; et al. The Origin of Highly Elevated Cell-Free DNA in Healthy Individuals and Patients with Pancreatic, Colorectal, Lung, or Ovarian Cancer. Cancer Discovery 2023, 13 (10), 2166–2179. 10.1158/2159-8290.CD-21-1252. 37565753 PMC10592331

[ref14] Chen Q.; Yin H.; Liu S.; Shoucair S.; Ding N.; Ji Y.; Zhang J.; Wang D.; Kuang T.; Xu X.; Yu J.; Wu W.; Pu N.; Lou W. Prognostic Value of Tumor-Associated N1/N2 Neutrophil Plasticity in Patients Following Radical Resection of Pancreas Ductal Adenocarcinoma. J. ImmunoTher. 2022, 10 (12), e005798 10.1136/jitc-2022-005798. PMC9730407 36600557

[ref15] Bonifay A.; Robert S.; Champagne B.; Petit P.-R.; Eugène A.; Chareyre C.; Duchez A.-C.; Vélier M.; Fritz S.; Vallier L.; Lacroix R.; Dignat-George F. A New Strategy to Count and Sort Neutrophil-Derived Extracellular Vesicles: Validation in Infectious Disorders. J. Extracell. Vesicles 2022, 11 (4), e12204 10.1002/jev2.12204. 35362257 PMC8971553

[ref16] Yu D.; Zhang J.; Wang M.; Ji R.; Qian H.; Xu W.; Zhang H.; Gu J.; Zhang X. Exosomal MiRNAs from Neutrophils Act as Accurate Biomarkers for Gastric Cancer Diagnosis. Clin. Chim. Acta 2024, 554, 117773 10.1016/j.cca.2024.117773. 38199579

[ref17] Zhang Q.; Jeppesen D. K.; Higginbotham J. N.; Franklin J. L.; Coffey R. J. Comprehensive Isolation of Extracellular Vesicles and Nanoparticles. Nat. Protoc. 2023, 18 (5), 1462–1487. 10.1038/s41596-023-00811-0. 36914899 PMC10445291

[ref18] Das S.; Lyon C. J.; Hu T. A Panorama of Extracellular Vesicle Applications: From Biomarker Detection to Therapeutics. ACS Nano 2024, 18 (14), 9784–9797. 10.1021/acsnano.4c00666. 38471757 PMC11008359

[ref19] Yu D.; Li Y.; Wang M.; Gu J.; Xu W.; Cai H.; Fang X.; Zhang X. Exosomes as a New Frontier of Cancer Liquid Biopsy. Mol. Cancer 2022, 21 (1), 56 10.1186/s12943-022-01509-9. 35180868 PMC8855550

[ref20] Meng Y.; Zhang Y.; Bühler M.; Wang S.; Asghari M.; Stürchler A.; Mateescu B.; Weiss T.; Stavrakis S.; deMello A. J. Direct Isolation of Small Extracellular Vesicles from Human Blood Using Viscoelastic Microfluidics. Sci. Adv. 2023, 9 (40), eadi5296 10.1126/sciadv.adi5296. 37801500 PMC10558121

[ref21] Zhuang J.; Xia L.; Zou Z.; Yin J.; Lin N.; Mu Y. Recent Advances in Integrated Microfluidics for Liquid Biopsies and Future Directions. Biosens. Bioelectron. 2022, 217, 114715 10.1016/j.bios.2022.114715. 36174359

[ref22] Lou C.; Yang H.; Hou Y.; Huang H.; Qiu J.; Wang C.; Sang Y.; Liu H.; Han L. Microfluidic Platforms for Real-Time in Situ Monitoring of Biomarkers for Cellular Processes. Adv. Mater. 2024, 36 (6), e2307051 10.1002/adma.202307051. 37844125

[ref23] Vyhlídalová Kotrbová A.; Gömöryová K.; Mikulová A.; Plešingerová H.; Sladeček S.; Kravec M.; Hrachovinová Š.; Potěšil D.; Dunsmore G.; Blériot C.; Bied M.; Kotouček J.; Bednaříková M.; Hausnerová J.; Minář L.; Crha I.; Felsinger M.; Zdráhal Z.; Ginhoux F.; Weinberger V.; et al. Proteomic Analysis of Ascitic Extracellular Vesicles Describes Tumour Microenvironment and Predicts Patient Survival in Ovarian Cancer. J. Extracell. Vesicles 2024, 13 (3), e12420 10.1002/jev2.12420. 38490958 PMC10942866

[ref24] Zheng L.; Wang H.; Zuo P.; Liu Y.; Xu H.; Ye B.-C. Rapid On-Chip Isolation of Cancer-Associated Exosomes and Combined Analysis of Exosomes and Exosomal Proteins. Anal. Chem. 2022, 94 (21), 7703–7712. 10.1021/acs.analchem.2c01187. 35575685

[ref25] Cheng W.; Yao Y.; Li D.; Duan C.; Wang Z.; Xiang Y. Asymmetrically Split DNAzyme-Based Colorimetric and Electrochemical Dual-Modal Biosensor for Detection of Breast Cancer Exosomal Surface Proteins. Biosens. Bioelectron. 2023, 238, 115552 10.1016/j.bios.2023.115552. 37542978

[ref26] Li H.; Chiang C.-L.; Kwak K. J.; Wang X.; Doddi S.; Ramanathan L. V.; Cho S. M.; Hou Y.-C.; Cheng T.-S.; Mo X.; Chang Y.-S.; Chang H.-L.; Cheng W.; Tsai W.-N.; Nguyen L. T. H.; Pan J.; Ma Y.; Rima X. Y.; Zhang J.; Reategui E.; et al. Extracellular Vesicular Analysis of Glypican 1 mRNA and Protein for Pancreatic Cancer Diagnosis and Prognosis. Adv. Sci. 2024, 11 (11), e2306373 10.1002/advs.202306373. PMC10953589 38204202

[ref27] Xie M.; Chen T.; Cai Z.; Lei B.; Dong C. A Digital Microfluidic Platform Coupled with Colorimetric Loop-Mediated Isothermal Amplification for On-Site Visual Diagnosis of Multiple Diseases. Lab Chip 2023, 23 (12), 2778–2788. 10.1039/D2LC01156E. 37195227

[ref28] Zhou S.; Hu T.; Han G.; Wu Y.; Hua X.; Su J.; Jin W.; Mou Y.; Mou X.; Li Q.; Liu S. Accurate Cancer Diagnosis and Stage Monitoring Enabled by Comprehensive Profiling of Different Types of Exosomal Biomarkers: Surface Proteins and MiRNAs. Small 2020, 16 (48), e2004492 10.1002/smll.202004492. 33174389

[ref29] Gurudatt N. G.; Gwak H.; Hyun K.-A.; Jeong S.-E.; Lee K.; Park S.; Chung M. J.; Kim S.-E.; Jo J. H.; Jung H.-I. Electrochemical Detection and Analysis of Tumor-Derived Extracellular Vesicles to Evaluate Malignancy of Pancreatic Cystic Neoplasm Using Integrated Microfluidic Device. Biosens. Bioelectron. 2023, 226, 115124 10.1016/j.bios.2023.115124. 36758487

[ref30] Li P.; Chen J.; Chen Y.; Song S.; Huang X.; Yang Y.; Li Y.; Tong Y.; Xie Y.; Li J.; Li S.; Wang J.; Qian K.; Wang C.; Du L. Construction of Exosome SORL1 Detection Platform Based on 3D Porous Microfluidic Chip and Its Application in Early Diagnosis of Colorectal Cancer. Small 2023, 19 (20), e2207381 10.1002/smll.202207381. 36799198

[ref31] Lu Y.; Ye L.; Jian X.; Yang D.; Zhang H.; Tong Z.; Wu Z.; Shi N.; Han Y.; Mao H. Integrated Microfluidic System for Isolating Exosome and Analyzing Protein Marker PD-L1. Biosens. Bioelectron. 2022, 204, 113879 10.1016/j.bios.2021.113879. 35180692

[ref32] Kwak T. J.; Nam Y. G.; Najera M. A.; Lee S. W.; Strickler J. R.; Chang W.-J. Convex Grooves in Staggered Herringbone Mixer Improve Mixing Efficiency of Laminar Flow in Microchannel. PLoS One 2016, 11 (11), e0166068 10.1371/journal.pone.0166068. 27814386 PMC5096722

[ref33] Zhang P.; Zhou X.; He M.; Shang Y.; Tetlow A. L.; Godwin A. K.; Zeng Y. Ultrasensitive Detection of Circulating Exosomes with a 3D-Nanopatterned Microfluidic Chip. Nat. Biomed. Eng. 2019, 3 (6), 438–451. 10.1038/s41551-019-0356-9. 31123323 PMC6556143

[ref34] Zhang Y.; Tong X.; Yang L.; Yin R.; Li Y.; Zeng D.; Wang X.; Deng K. A Herringbone Mixer Based Microfluidic Device HBEXO-Chip for Purifying Tumor-Derived Exosomes and Establishing MiRNA Signature in Pancreatic Cancer. Sens. Actuators B Chem. 2021, 332, 129511 10.1016/j.snb.2021.129511.

[ref35] De Felice M.; De Falco M.; Zappi D.; Antonacci A.; Scognamiglio V. Isothermal amplification-assisted diagnostics for COVID-19. Biosens. Bioelectron. 2022, 205, 114101 10.1016/j.bios.2022.114101. 35202984 PMC8849862

[ref36] Chen H.; Zhuang Z.; Chen Y.; Qiu C.; Qin Y.; Tan C.; Tan Y.; Jiang Y. A. Universal Platform for One-Pot Detection of Circulating Non-Coding RNA Bombining CRISPR-Cas12a and Branched Rolling Circle Amplification. Anal. Chim. Acta 2023, 1246, 340896 10.1016/j.aca.2023.340896. 36764778

[ref37] Wang R.; Zhao X.; Chen X.; Qiu X.; Qing G.; Zhang H.; Zhang L.; Hu X.; He Z.; Zhong D.; Wang Y.; Luo Y. Rolling Circular Amplification (RCA)-Assisted CRISPR/Cas9 Cleavage (RACE) for Highly Specific Detection of Multiple Extracellular Vesicle MicroRNAs. Anal. Chem. 2020, 92 (2), 2176–2185. 10.1021/acs.analchem.9b04814. 31875674

[ref38] Huang R.; He L.; Li S.; Liu H.; Jin L.; Chen Z.; Zhao Y.; Li Z.; Deng Y.; He N. A. Simple Fluorescence Aptasensor for Gastric Cancer Exosome Detection Based on Branched Rolling Circle Amplification. Nanoscale 2020, 12 (4), 2445–2451. 10.1039/C9NR08747H. 31894795

[ref39] Liu C.; Li B.; Lin H.; Yang C.; Guo J.; Cui B.; Pan W.; Feng J.; Luo T.; Chu F.; Xu X.; Zheng L.; Yao S. Multiplexed Analysis of Small Extracellular Vesicle-Derived mRNAs by Droplet Digital PCR and Machine Learning Improves Breast Cancer Diagnosis. Biosens. Bioelectron. 2021, 194, 113615 10.1016/j.bios.2021.113615. 34507095

[ref40] Veerman R. E.; Teeuwen L.; Czarnewski P.; Güclüler Akpinar G.; Sandberg A.; Cao X.; Pernemalm M.; Orre L. M.; Gabrielsson S.; Eldh M. Molecular Evaluation of Five Different Isolation Methods for Extracellular Vesicles Reveals Different Clinical Applicability and Subcellular Origin. J. Extracell. Vesicles 2021, 10 (9), e12128 10.1002/jev2.12128. 34322205 PMC8298890

[ref41] Tian F.; Zhang S.; Liu C.; Han Z.; Liu Y.; Deng J.; Li Y.; Wu X.; Cai L.; Qin L.; Chen Q.; Yuan Y.; Liu Y.; Cong Y.; Ding B.; Jiang Z.; Sun J. Protein Analysis of Extracellular Vesicles to Monitor and Predict Therapeutic Response in Metastatic Breast Cancer. Nat. Commun. 2021, 12 (1), 2536 10.1038/s41467-021-22913-7. 33953198 PMC8100127

[ref42] Xu H.; Wu X.; Liu Q.; Yang C.; Shen M.; Wang Y.; Liu S.; Zhao S.; Xiao T.; Sun M.; Ding Z.; Bao J.; Chen M.; Gao M. A Universal Strategy for eEnhancing the Circulating MiRNAs’ Detection Performance of Rolling Circle Amplification by Using a Dual-Terminal Stem-Loop Padlock. ACS Nano 2024, 18 (1), 436–450. 10.1021/acsnano.3c07721. 38149638 PMC10786163

[ref43] Weng W.-H.; Wang C.-Y.; Yan Z.-Y.; Lee H.-T.; Kao C.-Y.; Chang C.-W. Isolation and Characterizations of Multidrug-Resistant Human Cancer Cells by a Biodegradable Nano-Sensor. Biosens. Bioelectron. 2024, 249, 115985 10.1016/j.bios.2023.115985. 38219465

[ref44] Yang C.; Du C.; Yuan F.; Yu P.; Wang B.; Su C.; Zou R.; Wang J.; Yan X.; Sun C.; Li H. CRISPR/Cas12a-Derived Ratiometric Fluorescence Sensor for High-Sensitive Pb2+ Dtection Based on CDs@ZIF-8 and DNAzyme. Biosens. Bioelectron. 2024, 251, 116089 10.1016/j.bios.2024.116089. 38354496

[ref45] Hu S.; Zhang L.; Su Y.; Liang X.; Yang J.; Luo Q.; Luo H. Sensitive Detection of Multiple Blood Biomarkers via Immunomagnetic Exosomal PCR for the Diagnosis of Alzheimer’s Disease. Sci. Adv. 2024, 10 (13), eabm3088 10.1126/sciadv.abm3088. 38536917 PMC10971429

[ref46] Nakamura K.; Zhu Z.; Roy S.; Jun E.; Han H.; Munoz R. M.; Nishiwada S.; Sharma G.; Cridebring D.; Zenhausern F.; Kim S.; Roe D. J.; Darabi S.; Han I.-W.; Evans D. B.; Yamada S.; Demeure M. J.; Becerra C.; Celinski S. A.; Borazanci E.; et al. An Exosome-Based Transcriptomic Signature for Noninvasive, Early Detection of Patients with Pancreatic Ductal Adenocarcinoma: A Multicenter Cohort Study. Gastroenterology 2022, 163 (5), 1252–1266. 10.1053/j.gastro.2022.06.090. 35850192 PMC9613527

[ref47] Shin H.; Choi B. H.; Shim O.; Kim J.; Park Y.; Cho S. K.; Kim H. K.; Choi Y. Single Test-Based Diagnosis of Multiple Cancer Types Using Exosome-SERS-AI for Early-Stage Cancers. Nat. Commun. 2023, 14 (1), 1644 10.1038/s41467-023-37403-1. 36964142 PMC10039041

[ref48] Melo S. A.; Luecke L. B.; Kahlert C.; Fernandez A. F.; Gammon S. T.; Kaye J.; LeBleu V. S.; Mittendorf E. A.; Weitz J.; Rahbari N.; Reissfelder C.; Pilarsky C.; Fraga M. F.; Piwnica-Worms D.; Kalluri R. Glypican-1 Identifies Cancer Exosomes and Detects Early Pancreatic Cancer. Nature 2015, 523 (7559), 177–182. 10.1038/nature14581. 26106858 PMC4825698

