# Supplementary material for: Integrated Microfluidic Chip for Neutrophil Extracellular Vesicle Analysis and Gastric Cancer Diagnosis
Source: ACS Nano. 2025 Mar 10;19(10):10078–92. doi: 10.1021/acsnano.4c16894 (PMC11924328; doi:10.1021/acsnano.4c16894)
Supplement: Supplementary file 1 — nn4c16894_si_001.pdf [file nn4c16894_si_001.pdf]

SUPPORTING INFORMATION FOR

An integrated microfluidic chip for neutrophil extracellular vesicles analysis and gastric cancer diagnosis

Dan Yu[1,†], Jianmei Gu[2,†], Jiahui Zhang[1], Maoye Wang[1], Runbi Ji[1], Chunlai Feng[3], Hélder A. Santos[4,5,*], Hongbo Zhang[6,7,*], Xu Zhang[1,*]

[1]Jiangsu Key Laboratory of Medical Science and Laboratory Medicine, School of Medicine, Jiangsu University, Zhenjiang, Jiangsu 212013, China.

[2]Department of Clinical Laboratory Medicine, Affiliated Tumor Hospital of Nantong University, Nantong, Jiangsu 226361, China.

[3]School of Pharmacy, Jiangsu University, Zhenjiang, Jiangsu 212013, China.

[4]Department of Biomaterials and Biomedical Technology, University Medical Center Groningen, University of Groningen, Ant. Deusinglaan 1, Groningen 9713 AV , Netherlands.

[5]Drug Research Program, Division of Pharmaceutical Chemistry and Technology, Faculty of Pharmacy, University of Helsinki, FI-00014 Helsinki, Finland.

[6]Pharmaceutical Sciences Laboratory, Åbo Akademi University, Turku 20520, Finland.

[7]Turku Biosciences Center, University of Turku and Åbo Akademi University, Turku 20520, Finland.

† These authors contributed equally to this work.

*Corresponding author. Email: xuzhang@ujs.edu.cn (X.Z.); hongbo.zhang@abo.fi (H.Z.); h.a.santos@umcg.nl (H.A.S.)

This file includes:

Figure S1. Design principle of the IMCN chip.
Figure S2. Images of the developed IMCN chip.
Figure S3. Characterization of NEVs isolated by ultracentrifugation.
Figure S4. Characterization of aptamer binding activity.
Figure S5. Stability of the aptamers.
Figure S6. Verification of the superiority of IMCN chip for NEVs separation.
Figure S7. Characterization of the prepared LPs and RCA reaction.
Figure S8. Detection performance of RCA-MB assay using synthetic sequences.
Figure S9. Triplex amplification and detection of NEVs by RCA-MB assay.
Figure S10. Detection of serum NEVs derived miRNAs by RCA-MB assay.
Figure S11. The detection repeatability of the chip.
Figure S12. The diagnostic and prognostic performance of CEA and CA199.
Table S1. Comparison of the properties of NEVs isolated by ExoQuick, UC, and the microfluidic chip.
Table S2. All the nucleic acid sequences used in this study.
Table S3. The performance of NEVs miRNAs detected by dual RCA-MB assay in the differentiation and diagnosis of gastric cancer.
Table S4. The performance of NEVs miRNAs detected by dual RCA-MB assay in the diagnosis of gastric cancer of different stages.
Table S5. The performance of the NEV signatures detected by the microfluidic chip in the differentiation and diagnosis of gastric cancer.
Table S6. The performance of the NEV signatures detected by the microfluidic chip in the diagnosis of gastric cancer in different stages.
Table S7. The performance of conventional biomarkers (CEA, CA199) and their combinations (cCB), and combined four (cFB) or five biomarkers (cNCB) in the diagnosis of GC of different stages.
Table S8. The performance of single or combined biomarker with the assistance of ML in the diagnosis of gastric cancer.
Table S9. The confusion matrix, diagnostic sensitivity, specificity and accuracy of single or combined biomarker with the assistance of ML in the diagnosis of gastric cancer.
Table S10. The clinical information of samples used in RCA-MB assay for serum NEVs derived miRNAs detection.
Table S11. The clinical information of samples used in IMCN chip for serum NEVs detection.
Table S12. Comparison of EV detection performance reported in previous studies and our work.

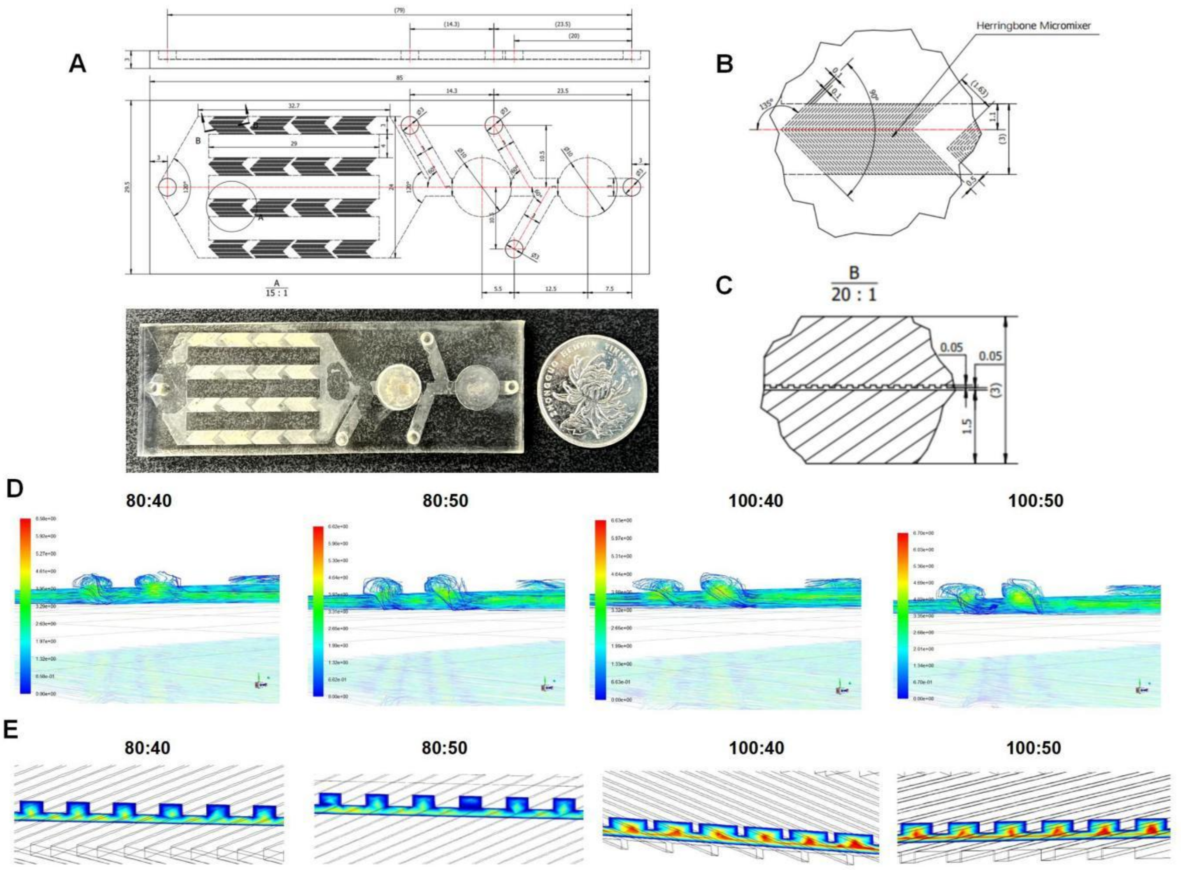

Figure S1. Design principle of the IMCN chip. (A) CAD drawing of the microfluidic chip. **(B-C)** The design of the asymmetrical herringbone grooves of the chip. **(D)** Fluid simulations of the flow velocity with different channel width to depth ratio (80:40, 80:50, 100:40, 100:50 μm). **(E)** Fluid simulations of the pressure profile with different channel width to depth ratio (80:40, 80:50, 100:40, 100:50 μm).

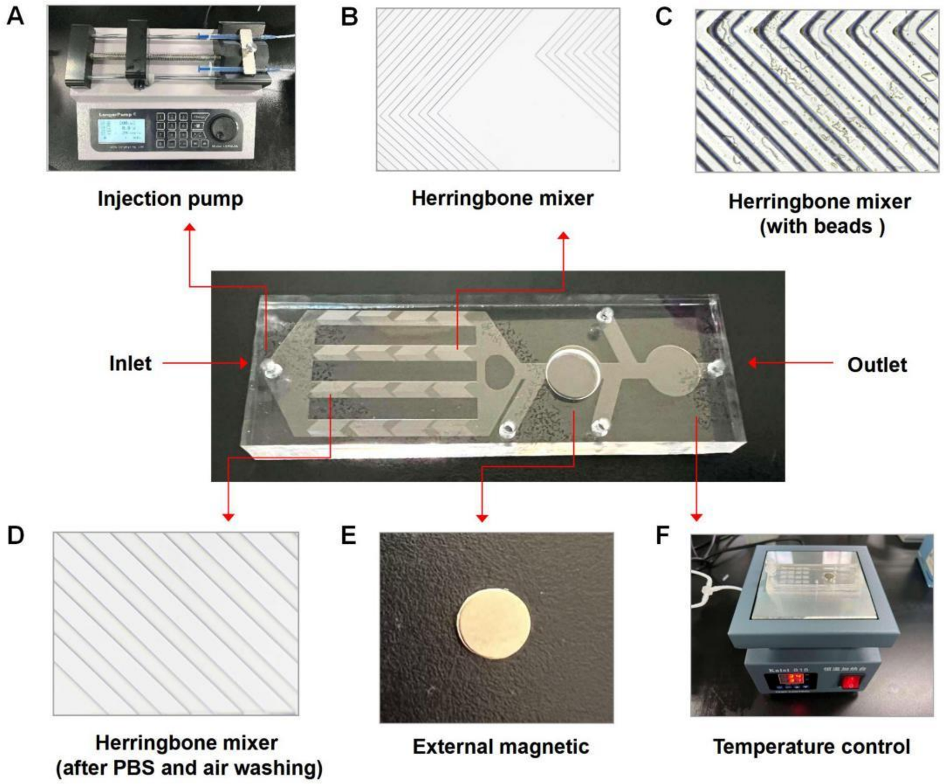

Figure S2. Images of the developed IMCN chip. (A) The image of the injection pump that used for fluid introducing. **(B)** The image of the asymmetrical herringbone mixer of the chip under microscope. **(C-D)** The image of herringbone mixer **(C)** with Dynabeads and **(D)** after PBS and air washing. **(E)** The image of external magnetic field used for NEVs capturing. **(F)** The image of temperature control machine that used for on-chip RCA-MB assay.

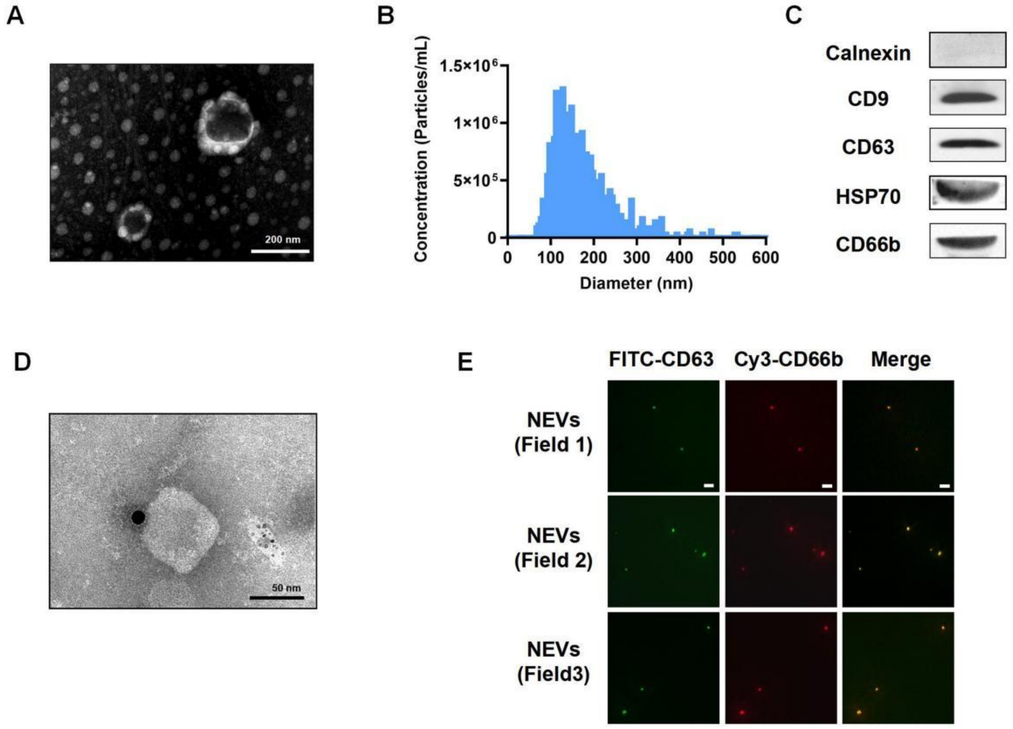

Figure S3. Characterization of NEVs isolated by ultracentrifugation. (A-C) Human peripheral blood derived NEVs were isolated by ultracentrifugation and characterized by **(A)** TEM (scale bar, 200 nm), **(B)** NTA and **(C)** western blot. **(D)** The location of CD66b on the surface of NEVs was verified by gold labeled immuno-transmission electron microscope (scale bar, 50 nm). **(E)** The colocalization of CD63 (green) and CD66b (red) on the surface of NEVs was detected by laser confocal fluorescence microscope (scale bar, 2 μm).

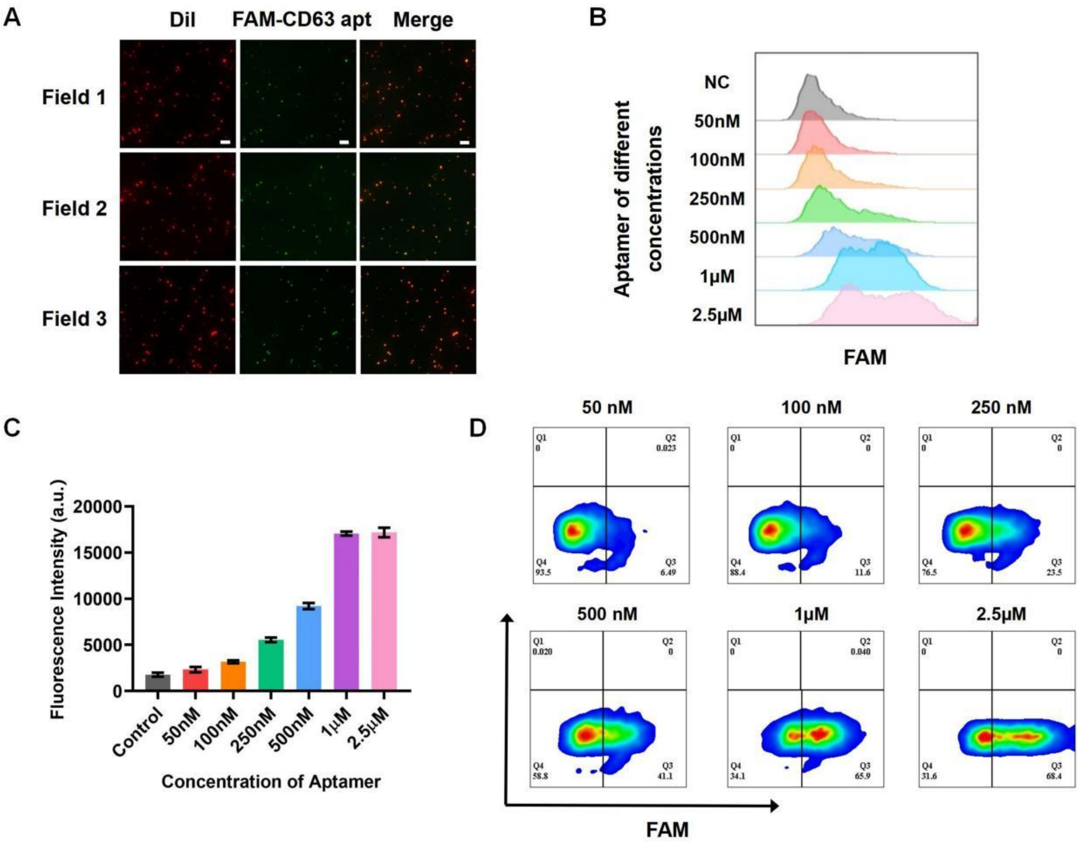

Figure S4. Characterization of aptamer binding activity. (A) Characterization of the binding of CD63 aptamers (green) to Dil-stained NEVs (red) by laser confocal fluorescence microscope (scale bar, 5 μm). **(B-C)** The binding activity of different concentrations of FAM-labeled CD63 aptamers (from 500 nM to 2.5 μM) to NEVs covalently coupled on aldehyde beads was determined by flow cytometry. **(D)** The binding efficiency of different concentrations of CD63 aptamers to NEVs.

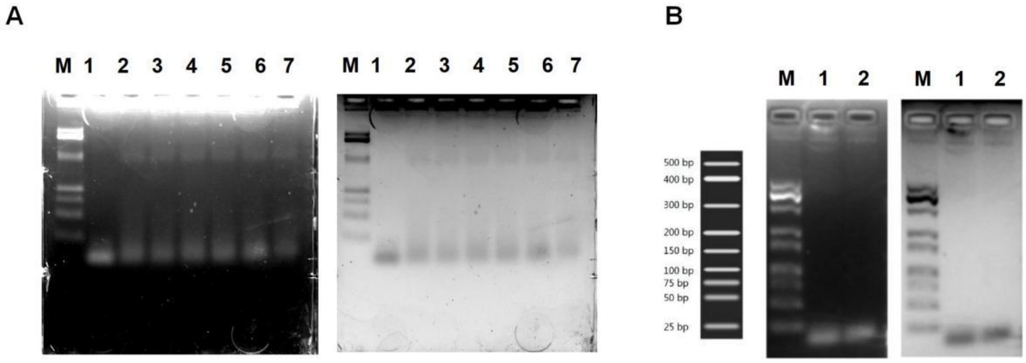

Figure S5. Stability of the aptamers. (A) The resistance of aptamers to nuclease degradation in human serum samples. Lane 1 to 7 indicated the aptamers incubated with 10% PBS diluted serum samples at 37 °C from 0 to 3 h, with an interval of 0.5 h. **(B)** The stability of aptamers in high temperature environment. Aptamers in room temperature (lane 1) and after 95 °C heating (lane 2).

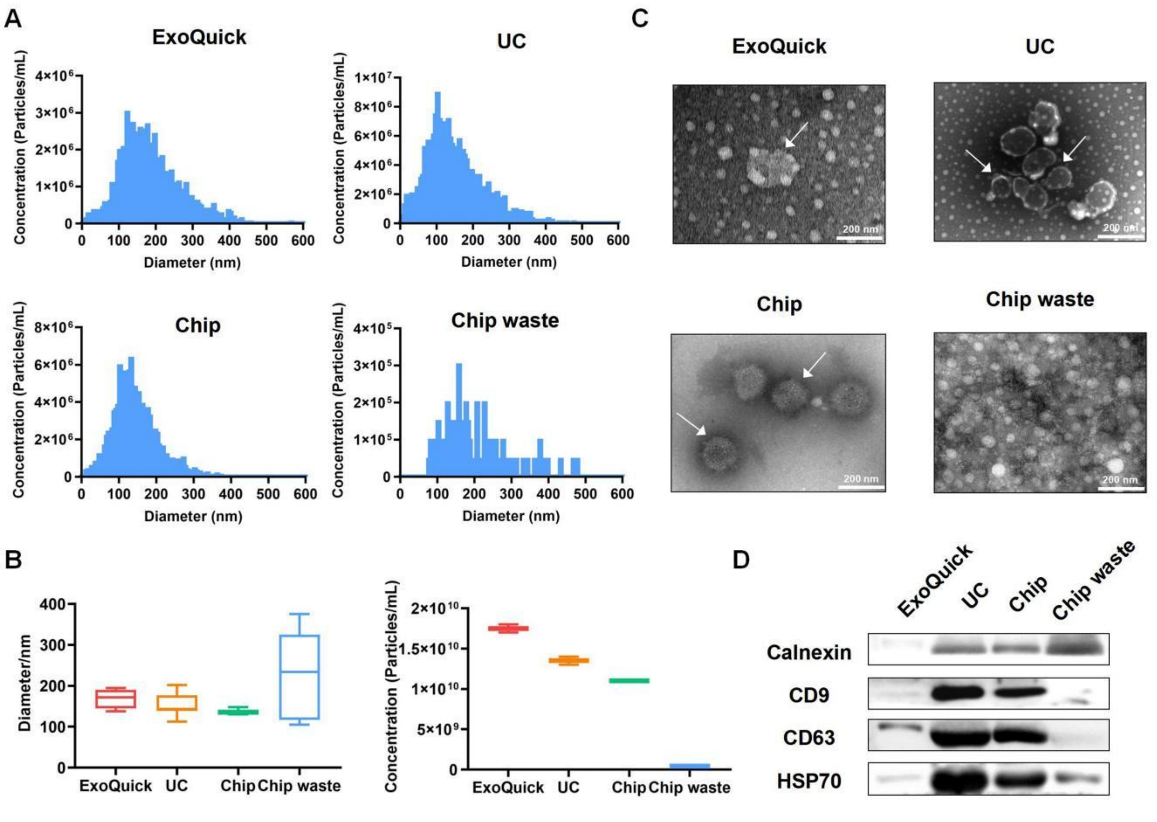

Figure S6. Verification of the superiority of IMCN chip for NEVs separation. (A) The particle size distribution of isolated NEVs in ExoQuick, UC, Chip and Chip waste groups. **(B)** The diameter and particle concentration of isolated NEVs in ExoQuick, UC, Chip and Chip waste groups. **(C)** TEM images of isolated NEVs in ExoQuick, UC, Chip and Chip waste groups (NEVs were pointed with white arrow, scale bar, 200 nm). **(D)** The expression of EVs markers in isolated NEVs of ExoQuick, UC, Chip and Chip waste groups.

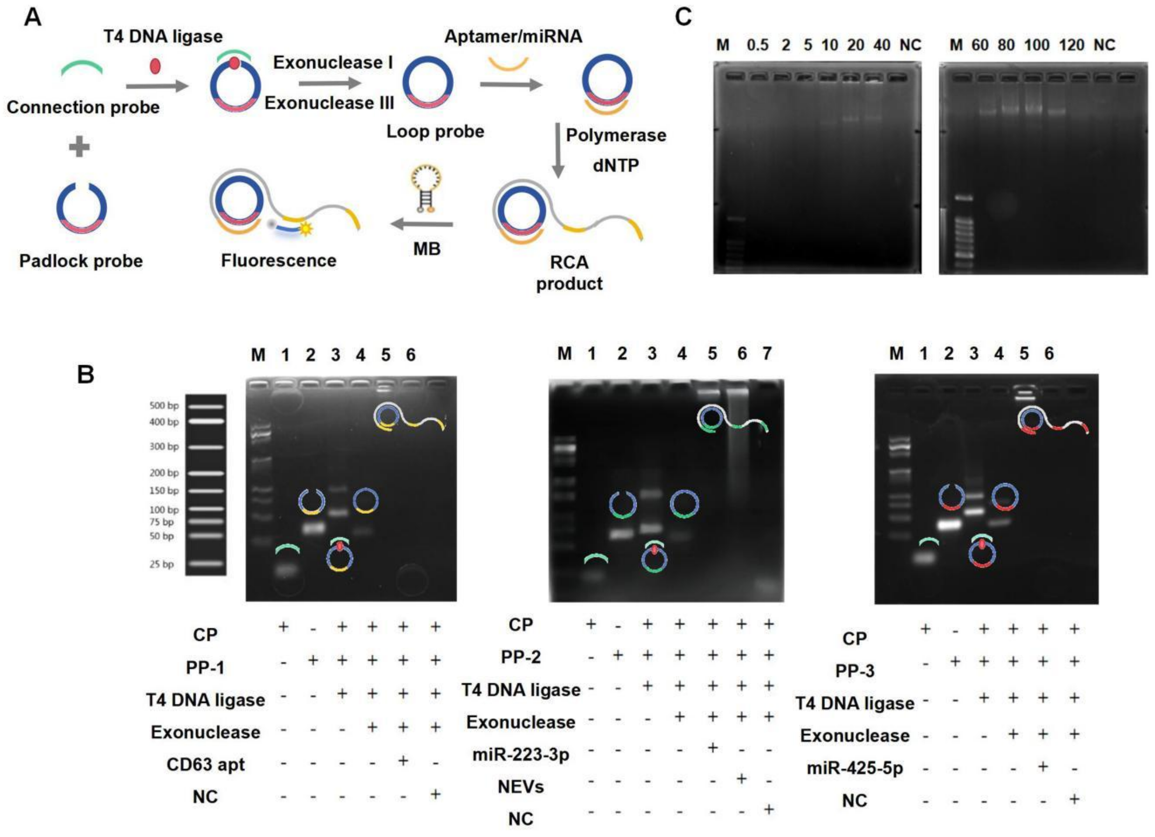

Figure S7. Characterization of the prepared LPs and RCA reaction. (A) Schematic illustration of the RCA-MB assay. **(B)** Preparation and characterization of the LPs by agarose gel electrophoresis. CP (lane 1), PP (lane 2), the product after connecting CP and PP by T4 DNA ligase (lane3), LP after Exonuclease treatment (lane 4) and the RCA products (lane 5). **(C)** Optimization of the amplification time for RCA reaction by agarose gel electrophoresis (from 0.5 to 120 min).

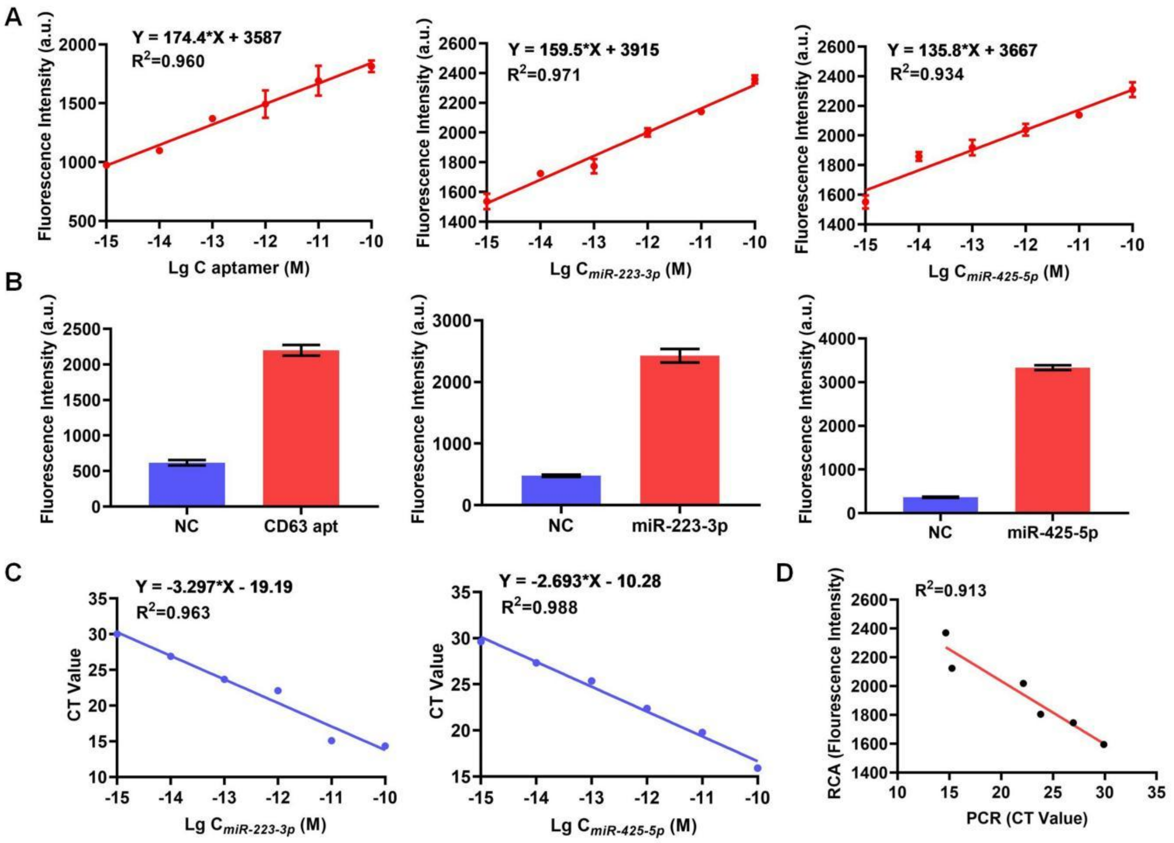

Figure S8. Detection performance of RCA-MB assay using synthetic sequences. (A) Linear range curve of the fluorescence intensities as detected by RCA-MB assay with different concentrations of aptamers and synthetic miRNAs. **(B)** Specificity of the RCA-MB assay. **(C)** Linear range curve of the CT value with different concentrations of synthesized miRNAs as detected by qRT-PCR. **(D)** Pearson correlation analysis of miRNA expression levels as detected by RCA-MBs assay and qRT-PCR.

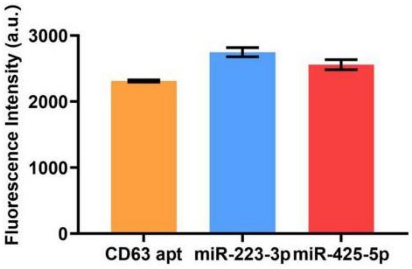

Figure S9. Triplex amplification and detection of NEVs by RCA-MB assay.

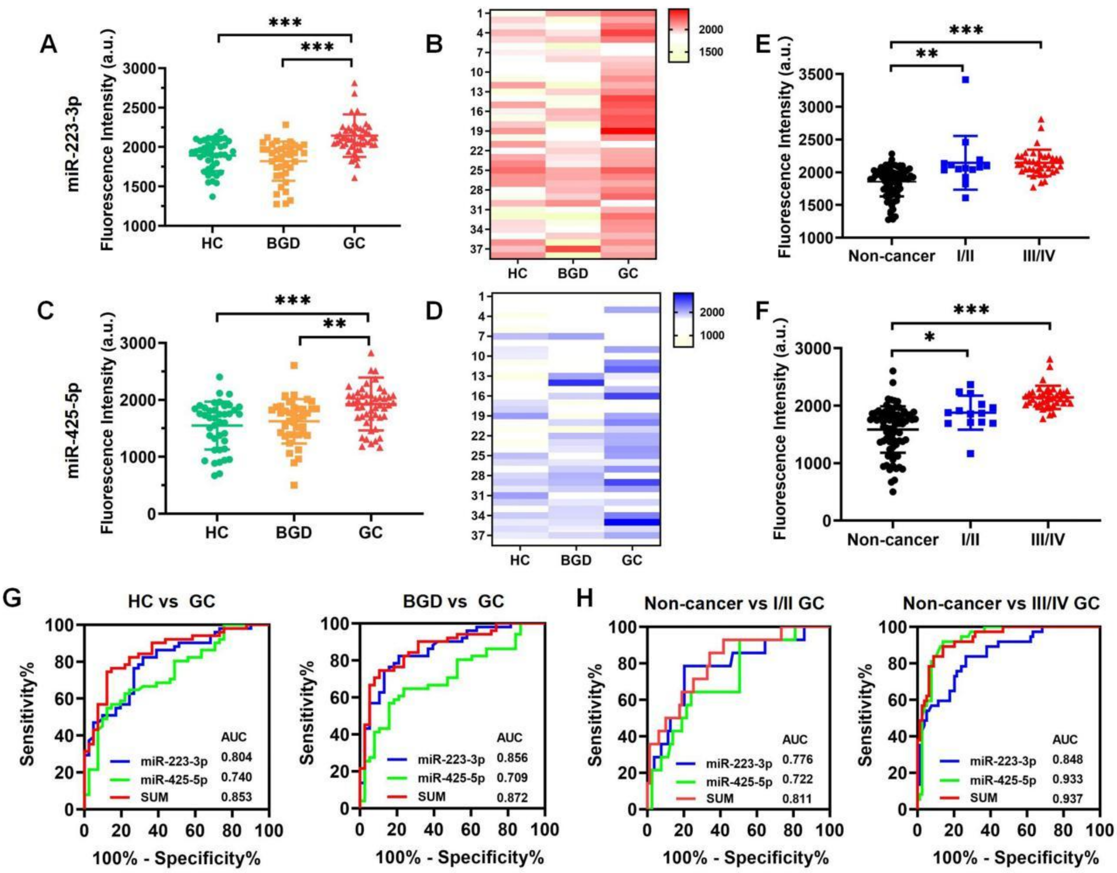

Figure S10. Detection of serum NEVs derived miRNAs by RCA-MB assay. (A-B) The expression of miR-223-3p in HC, BGD, and GC groups (****P<0.001*). **(C)** The expression of miR-223-3p in non-cancer groups and GC of different stages (****P<0.001*). **(D-E)** The expression of miR-425-5p in HC, BGD, and GC groups (****P<0.001*). **(F)** The expression of miR-425-5p in non-cancer groups and GC of different stages (****P<0.001*). **(G)** ROC curves of serum NEVs miRNAs in distinguishing between HC and GC patients, BGD and GC patients. **(H)** ROC curves of serum NEVs miRNAs in distinguishing between non-cancer groups and GC of different stages.

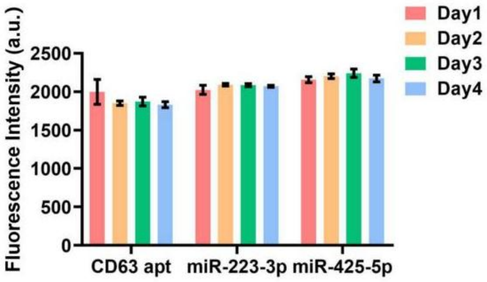

Figure S11. The detection repeatability of the chip. NEVs at a concentration of 10^9 EVs/mL were measured on chip 3 times independently on 4 different days.

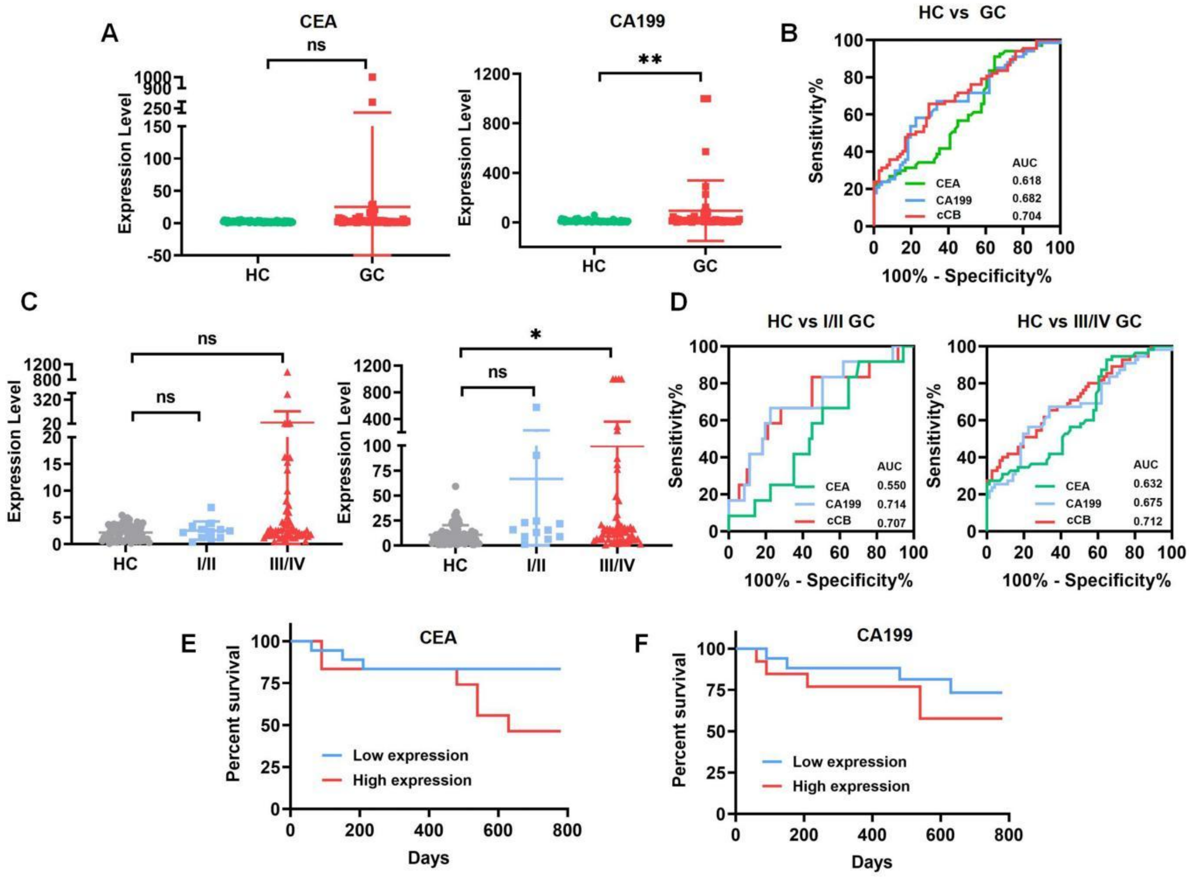

Figure S12. The diagnostic and prognostic performance of CEA and CA199. (A) The expression of CEA and CA199 in HC and GC groups. **(B)** ROC curves of CEA, CA199 and their combination (cCB) in distinguishing between HC and GC. **(C)** The expression of CEA and CA199 in HC and GC of different stages. **(D)** ROC curves of CEA, CA199s and their combination (cCB) in distinguishing between HC and GC of different stages. **(E-F)** The prognostic value of **(E)** CEA and **(F)** CA199 in GC patients.

Table S1. Comparison of the properties of NEVs isolated by ExoQuick, UC, and the microfluidic chip.

	ExoQuick	UC	Chip	Chip Waste
Diameter (nm)	169	154	138	223
Concentration (Particles/mL)	1.7×10^{10}	1.4×10^{10}	1.1×10^{10}	4.6×10^{8}

Table S2. All the nucleic acid sequences used in this study.

DNA or RNA	Sequence（5'→3'）
FAM-CD63 apt	FAM-CACCCCACCTCGCTCCCGTGACACTAATGCTA
CD63 apt	CACCCCACCTCGCTCCCGTGACACTAATGCTA[1]
miR-223-3p	UGUCAGUUUGUCAAAUACCCCA
miR-425-5p	AAUGACACGAUCACUCCCGUUGA
NC	UUGUACUACACAAAAGUACUG
CP	TGCCTACGTCAACAGCTCCAACTA CC
PP-1	P-TGACGTAGGCAAGATAGAGTAGCATTAGTGTCACGGGAGCGAG GTGGGGTGTCGTAGGACT TAAAGGTAGTTGGAGCTGT
PP-2	P-TGACGTAGGCAAGATAGAGTGGGGTATTTGACAAACTGACATC GTAGGACTTAAAGGTAGTTGGAGCTGT
PP-3	P-TGACGTAGGCAAGATAGAGTCAACGGGAGTGATCGTGTCATTT CGTAGGACTTAAAGGTAGTTGGAGCTGT
MB-1	CY5-CGCACCCACCCCACCTCGCTCCCGTGACACTAATGCTAGGTG CG-BHQ2
MB-2	FAM-CGCACCTGGGGTATTTGACAAACTGACAGGTGCG-Dabcyl
MB-3	Cy3-CGCACCTCAACGGGAGTGATCGTGTCATTGGTGCG-BHQ2

Table S3. The performance of NEVs miRNAs detected by dual RCA-MB assay in the differentiation and diagnosis of gastric cancer.

	HC vs GC			BGD vs GC		
	Sensitivity%	Specificity%	AUC	Sensitivity%	Specificity%	AUC
MiR-223-3p	82.35	68.29	0.804	74.51	86.84	0.856
MiR-425-5p	54.90	87.80	0.740	56.86	84.21	0.709
SUM	74.51	87.80	0.853	74.51	89.47	0.872

Table S4. The performance of NEVs miRNAs detected by dual RCA-MB assay in the diagnosis of gastric cancer of different stages.

	Non-cancer vs I/II stages GC			Non-cancer vs III/IV stages GC		
	Sensitivity%	Specificity%	AUC	Sensitivity%	Specificity%	AUC
MiR-223-3p	78.57	79.75	0.776	83.78	73.42	0.848
MiR-425-5p	92.86	49.37	0.722	91.89	86.08	0.933
SUM	85.71	65.82	0.811	89.19	86.08	0.937

Table S5. The performance of the NEV signatures detected by the microfluidic chip in the differentiation and diagnosis of gastric cancer.

	HC vs GC			BGD vs GC		
	Sensitivity%	Specificity%	AUC	Sensitivity%	Specificity%	AUC
CD66b/CD63$^+$ EVs	83.58	60.56	0.790	83.58	48.00	0.623
MiR-223-3p	82.09	80.28	0.861	74.63	86.00	0.850
MiR-425-5p	74.63	84.51	0.853	49.25	84.00	0.707
NEV signatures	83.58	84.51	0.891	70.15	88.00	0.857

NEV signatures: a combined analysis of three NEV biomarkers (CD66b/CD63$^+$ EVs, miR-223-3p and miR-425-5p).

Table S6. The performance of the NEV signatures detected by the microfluidic chip in the diagnosis of gastric cancer in different stages.

	Non-cancer vs I/II stages GC			Non-cancer vs III/IV stages GC		
	Sensitivity%	Specificity%	AUC	Sensitivity%	Specificity%	AUC
CD66b/CD63$^+$ EVs	100.00	32.23	0.642	87.27	55.37	0.742
MiR-223-3p	83.33	70.25	0.792	78.18	85.12	0.851
MiR-425-5p	58.33	80.17	0.657	78.18	71.90	0.808
NEV signatures	58.33	88.43	0.768	80.00	85.12	0.895

NEV signatures: a combined analysis of three NEV biomarkers (CD66b/CD63$^+$ EVs, miR-223-3p and miR-425-5p).

Table S7. The performance of conventional biomarkers (CEA, CA199) and their combinations (cCB), and combined four (cFB) or five biomarkers (cNCB) in the diagnosis of GC of different stages.

	HC vs GC			HC vs I/II stages of GC			HC vs III/IV stages of GC		
	Sen%	Spe%	AUC	Sen%	Spe%	AUC	Sen%	Spe%	AUC
CEA	91.04	35.21	0.618	91.67	29.58	0.550	92.73	35.21	0.632
CA199	58.21	77.46	0.682	66.67	77.46	0.714	56.36	77.46	0.675
cCB	65.67	70.42	0.704	66.67	71.83	0.707	65.45	66.20	0.712
cFB	73.13	92.96	0.894	66.67	91.55	0.823	76.36	91.55	0.907
cNCB	82.09	87.31	0.912	66.67	92.96	0.815	90.91	81.69	0.933

Table S8. The performance of single or combined biomarker with the assistance of ML in the diagnosis of gastric cancer.

	HC vs GC				HC vs GC		
	Sen%	Spe%	AUC		Sen%	Spe%	AUC
CD66b/CD63^{+} EVs	55.00	59.09	0.50	CEA	80.00	68.18	0.78
MiR-223-3p	90.00	86.36	0.89	CA199	55.00	77.27	0.67
MiR-425-5p	75.00	86.36	0.90	cCB	75.00	59.09	0.76
NEV signatures	80.00	81.81	0.88	cFB	85.00	86.36	0.89
cNCB	90.00	86.36	0.91				

Table S9. The confusion matrix, diagnostic sensitivity, specificity and accuracy of single or combined biomarker with the assistance of ML in the diagnosis of gastric cancer.

Biomarkers			Predicted		Sensitivity%	Specificity%	Accuracy%
CD66b/CD63^{+} EVs			HC	GC			
	Observed	HC	13	9	55.00	59.09	57.14
		GC	9	11			
MiR-223-3p			Predicted		Sensitivity%	Specificity%	Accuracy%
			HC	GC			
	Observed	HC	19	3	90.00	86.36	88.09
		GC	2	18			
MiR-425-5p			Predicted		Sensitivity%	Specificity%	Accuracy%
			HC	GC			
	Observed	HC	19	3	75.00	86.36	80.95
		GC	5	15			
NEV signatures			Predicted		Sensitivity%	Specificity%	Accuracy%
			HC	GC			
	Observed	HC	18	4	80.00	81.81	80.95
		GC	4	16			
CEA			Predicted		Sensitivity%	Specificity%	Accuracy%
			HC	GC			
	Observed	HC	15	7	80.00	68.18	73.81
		GC	4	16			
CA199			Predicted		Sensitivity%	Specificity%	Accuracy%
			HC	GC			
	Observed	HC	17	5	55.00	77.27	66.67
		GC	9	11			
cCB			Predicted		Sensitivity%	Specificity%	Accuracy%
			HC	GC			
	Observed	HC	13	9	75.00	59.09	66.67
		GC	5	15			
cFB			Predicted		Sensitivity%	Specificity%	Accuracy%
			HC	GC			
	Observed	HC	19	3	85.00	86.36	85.71
		GC	3	17			
cNCB			Predicted		Sensitivity%	Specificity%	Accuracy%
			HC	GC			
	Observed	HC	19	3	90.00	86.36	88.10
		GC	2	18			

Table S10. The clinical information of samples used in RCA-MB assay for serum NEVs derived miRNAs detection.

HC			BGD			GC			
	**Gender**	**Age**		**Gender**	**Age**		**Gender**	**Age**	**TNM stage**
1	Male	72	1	Female	57	1	Male	73	T2N1M0
2	Male	56	2	Female	71	2	Male	53	T2N1M0
3	Male	62	3	Female	76	3	Female	76	T4bN3M1
4	Male	71	4	Female	76	4	Male	56	T4N1M0
5	Male	58	5	Female	71	5	Male	64	T4bN1M0
6	Male	73	6	Female	68	6	Male	67	T3N1M0
7	Male	73	7	Female	59	7	Male	73	T4aN1M0
8	Male	63	8	Female	63	8	Male	54	T3N0M0
9	Male	71	9	Female	68	9	Female	67	T4N3M1
10	Female	54	10	Male	67	10	Male	70	T1N0M0
11	Male	58	11	Male	64	11	Female	56	T4N1M1
12	Female	67	12	Female	83	12	Male	86	T2N1M0
13	Female	56	13	Female	68	13	Male	71	T4N1Mx
14	Male	55	14	Female	53	14	Male	70	T4aN1M0
15	Male	60	15	Female	67	15	Male	69	T4aN1M0
16	Male	72	16	Female	68	16	Female	69	T4aN1M0
17	Male	67	17	Female	48	17	Female	60	T4bN1M0
18	Male	57	18	Female	65	18	Male	54	T3N1Mx
19	Male	51	19	Female	70	19	Male	70	T3N1Mx
20	Male	56	20	Female	70	20	Male	67	T2N0M0
21	Male	85	21	Female	60	21	Male	68	T2N1Mx
22	Male	53	22	Male	70	22	Male	69	T2N0M0
23	Female	76	23	Male	67	23	Female	66	T2N0M0
24	Male	53	24	Female	49	24	Male	50	T4aN2M0
25	Male	53	25	Female	60	25	Male	77	T3N1M0
26	Male	54	26	Male	66	26	Male	67	T4aN2M0
27	Male	56	27	Male	72	27	Female	51	T4aN2M0
28	Male	54	28	Female	63	28	Female	67	T2N0M0
29	Male	52	29	Male	58	29	Male	74	T4aN2M0
30	Male	50	30	Female	58	30	Male	76	T4aN1M0
31	Female	74	31	Female	61	31	Female	77	T4aN2M0
32	Female	69	32	Female	70	32	Male	66	T4aN2M0

33	Female	74	33	Female	70	33	Male	63	T3N0M0
34	Male	71	34	Female	65	34	Female	75	T2N1M0
35	Male	73	35	Female	49	35	Male	59	T4aN1M0
36	Male	51	36	Female	60	36	Female	73	T2N1M2
37	Female	72	37	Female	48	37	Male	77	T3N2M0
38	Female	72	38	Female	68	38	Male	72	T2N1M0
39	Female	72				39	Male	67	T4aN1M0
40	Female	73				40	Male	77	T3N1M0
41	Female	75				41	Female	76	T2N0M0
						42	Male	65	T3N1M0
						43	Female	79	T3N1M0
						44	Male	58	T1aN0M0
						45	Male	65	T4aN3M1
						46	Male	66	T4aN3M1
						47	Male	63	T4aN1M0
						48	Female	70	T4bN2M0
						49	Female	74	T4aN3M0
						50	Male	62	T3N1M0
						51	Female	59	T4aN2M0

Table S11. The clinical information of samples used in IMCN chip for serum NEVs detection.

HC					BGD		
	Gender	Age	CEA	CA199		Gender	Age
1	Male	68	1.31	10.5	1	Male	69
2	Male	50	2.13	9.31	2	Male	42
3	Male	22	3.11	5.74	3	Male	72
4	Male	45	0.65	12.9	4	Male	67
5	Male	66	0.94	9.88	5	Male	55
6	Male	65	0.65	7.42	6	Female	68
7	Male	60	0.41	12.1	7	Female	50
8	Male	65	2.05	18.8	8	Male	41
9	Male	56	0.54	18	9	Male	58
10	Male	66	0.78	1.32	10	Female	58
11	Male	34	0.71	1	11	Female	61
12	Male	52	0.15	10.8	12	Female	70
13	Male	51	3.21	5.8	13	Female	70
14	Male	58	2.89	6.05	14	Female	65
15	Male	62	2.14	12.6	15	Female	49
16	Male	59	2.07	11.5	16	Female	60
17	Male	69	1.14	9.45	17	Female	48
18	Male	54	2.51	9.41	18	Female	68
19	Male	68	3.19	12.5	19	Female	46
20	Male	54	2.22	4.93	20	Male	72
21	Male	48	2.19	8.3	21	Male	62
22	Male	57	0.69	10.6	22	Female	63
23	Male	56	3.57	1.00	23	Female	60
24	Male	60	2.69	5.75	24	Male	66
25	Female	58	3.54	9.66	25	Male	75
26	Female	45	3.11	6.12	26	Male	70
27	Female	58	3.97	2.46	27	Male	67
28	Female	70	2.72	1.28	28	Female	49
29	Female	56	3.14	1.05	29	Female	68
30	Female	69	0.46	0.97	30	Female	48
31	Female	60	0.31	4.91	31	Female	65
32	Female	53	4.61	2.85	32	Female	70
33	Female	50	0.99	12.97	33	Female	70
34	Female	60	1.54	2.54	34	Female	83
35	Female	53	2.31	8.2	35	Female	60

36	Female	53	3.55	21.5	36	Female	68
37	Female	61	2.68	1.03	37	Female	53
38	Female	64	5.4	30.2	38	Female	67
39	Female	52	4.11	23.9	39	Female	57
40	Female	49	2.49	4.24	40	Female	71
41	Female	49	1.64	1.63	41	Female	76
42	Female	58	1.17	1.14	42	Female	76
43	Female	52	2.64	3.5	43	Female	71
44	Female	59	2.98	10.5	44	Female	68
45	Female	64	3.16	5.2	45	Female	59
46	Female	51	0.88	3.3	46	Female	63
47	Female	60	3.73	8.4	47	Female	68
48	Female	55	4.12	7.9	48	Male	67
49	Female	54	0.17	6.1	49	Male	64
50	Female	56	2.11	3.8	50	Male	78
51	Female	54	4.91	25.2			
52	Male	61	0.79	1.81			
53	Female	59	4.35	27.7			
54	Male	72	0.75	10.7			
55	Female	69	0.64	14.4			
56	Female	69	0.55	14.4			
57	Female	51	4.33	7.04			
58	Male	69	1.27	8.51			
59	Female	57	2.19	26.4			
60	Female	73	2.87	24			
61	Male	66	0.38	7.71			
62	Male	50	4.15	12.8			
63	Female	61	0.95	10.4			
64	Female	52	3.44	21.5			
65	Male	55	0.61	4.38			
66	Female	65	0.45	11.7			
67	Female	65	3.65	33.3			
68	Female	67	3.94	15.7			
69	Female	65	1.1	59.3			
70	Female	50	2.45	10.9			
71	Female	59	1.86	17.5			

GC					
	**Gender**	**Age**	**TNM stage**	**CEA**	**CA199**
1	Female	55	T3N1M0	1.11	11.84
2	Male	63	T4N1M0	1.63	16.45
3	Male	71	T3N2M0	1.77	17.97
4	Male	51	T2N0M0	2.36	1.33
5	Male	52	T4N1M0	9.99	0.8

6	Male	48	T3N1M0	1.18	8.79
7	Male	68	T4N1M0	27.35	22.87
8	Female	79	T4aN2M0	2.45	14.53
9	Male	84	T2N1M0	6.84	90.34
10	Female	69	T3N1M0	3.21	18.69
11	Male	59	T4N1M1	1.57	5.91
12	Male	77	T3N2M0	3.97	31.09
13	Male	77	T2N0M0	2.73	22.9
14	Female	66	T4N3M1	1.18	13.5
15	Male	74	T3N2M0	2.34	76.47
16	Male	84	T4aN2M0	21.11	3.4
17	Male	64	T4aN2M1	2.88	1.77
18	Male	79	T4N3M0	2.56	4.58
19	Male	63	T4N1M0	2.13	2.86
20	Male	83	T4N1M1	388.4	289
21	Male	78	T3N3aM0	2.59	16.89
22	Male	64	T3N0M0	5.17	16.76
23	Male	64	T3N2M0	2.91	6.96
24	Male	80	T3N1M1	15.33	1000
25	Female	44	T2N0M0	0.99	24.51
26	Male	85	T4N1M0	1.98	16.52
27	Male	78	T4N1M0	1.76	11.36
28	Male	66	T2N1M1	1.21	8.9
29	Female	62	T3N2M0	1.8	6.66
30	Male	69	T3N3bM0	0.58	5.26
31	Female	49	T3N0M0	1.56	16.82
32	Male	61	T1N0M0	2.48	13.2
33	Male	78	T4N1M0	1.79	49.16
34	Male	56	T3N2M0	1.58	6.7
35	Male	73	T3N2M0	2.58	17
36	Male	84	T4aN2M1	1.36	87.4
37	Male	78	T4aN2M0	3.6	6.43
38	Male	71	T4N1M0	0.71	4.85
39	Male	79	T4N3M1	1.64	11.7
40	Female	51	T3N3M1	2.18	123
41	Female	60	T2N0M0	0.39	15.6
42	Male	63	T2N1M0	3.37	15.8
43	Female	68	T2N1M1	2.18	21.8
44	Male	38	T3N3M0	1.93	14.9
45	Male	70	T4N1M1	5.98	13.4
46	Male	70	T3N0M0	16.3	28.3
47	Male	76	T3NxM1	1.77	6.21
48	Male	71	T2N1M0	2.76	572
49	Male	74	T4NxM1	4.15	15.6

50	Female	31	T4N4M1	7.28	2
51	Male	74	T4aN2M0	8.09	81.2
52	Male	51	T3N2M0	0.53	6.2
53	Male	77	T4aN2M1	6.23	19.4
54	Female	59	T4aN2M1	16.2	226
55	Male	72	T3N2M0	1.93	11
56	Male	72	T2N0M0	1.21	8.9
57	Female	56	T3N1M0	2.58	45.1
58	Male	66	T3N2M1	4.13	11.3
59	Female	72	T4N1M1	2.58	17
60	Female	54	T4N3M0	2.51	1000
61	Male	88	T3N2M1	29.2	1000
62	Male	70	T3N3aM0	2.89	5.87
63	Male	51	T4N3M1	1000	1000
64	Male	57	T4N3M1	13.9	12.1
65	Male	60	T3N1M0	1.48	5
66	Female	53	T4aN2M0	1.21	20.9
67	Female	62	T2N1M1	3.92	6.16

Table S12. Comparison of EV detection performance reported in previous studies and our work.

Detection platform	Sample volume	Detected EV type	Biomarkers	Isolation method	Amplified method	Detection method	Signal reading	Operation time	Diagnostic model	Diagnostic performance	Ref
Apt-Fusion	5 µL	PD-L1 positive EVs	miR-21	Aptamer decorated liposome	Without amplification	Molecular beacon	Flow cytometry	More than 2 h	Not applied	AUC of 0.9551	[2]
iMER assay	100 µL	Tumour exosomes	EPHA2, EGFR and PDPN	Immuno-magnetic capture	Reverse transcription and qPCR		qPCR	~2 h	Not applied	Accuracy of 90%	[3]
SORTER	0.2 µL	Tumor derived exosomes	miR-222, miR-1290, miR-182, miR-21, miR-221, and miR-10b	Aptamer decorated liposome	DSN	Au NFs and DNA FAM	Microplate reader	~2 h	LDA	Accuracy of 90.6%	[1]
Microfluidic coflow system	Not mention ed	HER2/ EpCAM positive EV	HER2/ EpCAM	λ-DNA	Without amplification	Aptamer	Fluorescence microscopy	Not mentioned	LDA	Not mentioned	[4]
IMCN	10 µL	NEVs	CD66b/CD63$^+$ NEVs, miR-223-3p and miR-425-5p	Immuno-magnetic capture	RCA	Molecular beacons	Microplate reader	Less than 4 h	RF	AUC of 0.91	Our work

Reference:

[1] Lei, Y.; Fei, X.; Ding, Y.; Zhang, J.; Zhang, G.; Dong, L.; Song, J.; Zhuo, Y.; Xue, W.; Zhang, P.; Yang, C. Simultaneous Subset Tacing and MiRNA Profiling of Tumor-Derived Exosomes via Dual-Surface-Protein Orthogonal Barcoding. *Sci. Adv.* **2023**, 9 (40), eadi1556.

[2] Cui, L.; Peng, R.; Zeng, C.; Zhang, J.; Lu, Y.; Zhu, L.; Huang, M.; Tian, Q.; Song, Y.; Yang, C. A General Strategy for Detection of Tumor-Derived Extracellular Vesicle MicroRNAs Using Aptamer-Mediated Vesicle Fusion. *Nano Today* **2022**, 46, 101599.

[3] Shao, H.; Chung, J.; Lee, K.; Balaj, L.; Min, C.; Carter, B. S.; Hochberg, F. H.; Breakefield, X. O.; Lee, H.; Weissleder, R. Chip-Based Analysis of Exosomal mRNA Mediating Drug Resistance in Glioblastoma. *Nat. Commun.* **2015**, 6, 6999.

[4] Liu, C.; Zhao, J.; Tian, F.; Chang, J.; Zhang, W.; Sun, J. λ-DNA- and Aptamer-Mediated Sorting and Analysis of Extracellular Vesicles. *J. Am. Chem. Soc.* **2019**, 141 (9), 3817-3821.
